# Genomic fossils reveal adaptation of non-autonomous pararetroviruses driven by concerted evolution of noncoding regulatory sequences

**DOI:** 10.1371/journal.ppat.1006413

**Published:** 2017-06-29

**Authors:** Sunlu Chen, Huizhen Zheng, Yuji Kishima

**Affiliations:** Laboratory of Plant Breeding, Research Faculty of Agriculture, Hokkaido University, Sapporo, Japan; University of Glasgow MRC Virology Unit, UNITED KINGDOM

## Abstract

The interplay of different virus species in a host cell after infection can affect the adaptation of each virus. Endogenous viral elements, such as endogenous pararetroviruses (PRVs), have arisen from vertical inheritance of viral sequences integrated into host germline genomes. As viral genomic fossils, these sequences can thus serve as valuable paleogenomic data to study the long-term evolutionary dynamics of virus–virus interactions, but they have rarely been applied for this purpose. All extant PRVs have been considered autonomous species in their parasitic life cycle in host cells. Here, we provide evidence for multiple non-autonomous PRV species with structural defects in viral activity that have frequently infected ancient grass hosts and adapted through interplay between viruses. Our paleogenomic analyses using endogenous PRVs in grass genomes revealed that these non-autonomous PRV species have participated in interplay with autonomous PRVs in a possible commensal partnership, or, alternatively, with one another in a possible mutualistic partnership. These partnerships, which have been established by the sharing of noncoding regulatory sequences (NRSs) in intergenic regions between two partner viruses, have been further maintained and altered by the sequence homogenization of NRSs between partners. Strikingly, we found that frequent region-specific recombination, rather than mutation selection, is the main causative mechanism of NRS homogenization. Our results, obtained from ancient DNA records of viruses, suggest that adaptation of PRVs has occurred by concerted evolution of NRSs between different virus species in the same host. Our findings further imply that evaluation of within-host NRS interactions within and between populations of viral pathogens may be important.

## Author summary

This paper addresses the adaptive strategies of ancient defective viruses recorded in grass genomes. We mined numerous virus segments from various grass genomes and assembled several defective pararetrovirus (non-autonomous PRV) species. We attempted to understand how these non-autonomous PRVs can complete parasitic life cycles in host plants. We determined that these non-autonomous PRV species have participated in interplay with autonomous PRVs or different non-autonomous PRV species. This interplay between different virus genomes has involved the exchange of noncoding regulatory sequences, which consequently evolved to be extraordinarily highly similar in different viruses within the same host. In non-autonomous PRVs, adaptive strategies to compensate for a lack of functionality have consequently involved concerted evolution of noncoding sequences establishing the partnerships.

## Introduction

Similar to virus–host interactions, virus–virus interactions, especially those occurring during mixed plant virus infections in nature, have complex outcomes ranging from antagonism to synergism [1, 2]. Such interactions between different virus species affect their adaptation [1, 2]. Numerous virus-derived sequences, referred to as endogenous viral elements (EVEs), have recently been discovered in various eukaryotic genomes [3–6]. In addition to EVEs derived from retroviruses, EVEs originating from viruses without active reverse-transcription or integration abilities have been identified [4, 7–10]. Because these elements are vertically inherited viral sequences integrated into the germline genome of a host, they are viral genomic fossils and hence serve as invaluable historical records [3, 11, 12]. Although EVEs may provide an unprecedented opportunity to advance our understanding of evolutionary-scale virus–virus interactions, these records have rarely been exploited to explore such interactions.

Pararetroviruses (PRVs), including *Caulimoviridae* and *Hepadnaviridae* families, are reverse-transcribing double-stranded DNA viruses that lack an integrase and a process for integration [5, 13]. PRVs also possess EVEs called endogenous PRVs that originated from the incidental integration of PRV DNA into host genomes through non-homologous end-joining [14, 15]. Endogenous PRVs have been identified in an increasing number of plant genomes and have also been recently discovered in bird and reptile genomes [4, 5, 11, 16–18].

PRVs are thought to be distantly related to long terminal repeat (LTR) retrotransposons [19]. Interestingly, many LTR retrotransposons are non-autonomous with respect to their parasitic life cycle in host cells, i.e., they have lost most or all of their coding capability but can amplify themselves by using the protein machinery of autonomous LTR retrotransposons that are functionally and structurally intact [20–22]. A hallmark of the parasitism of non-autonomous LTR retrotransposons on their autonomous partners is the substantial sequence similarity of their LTRs—the location of noncoding regulatory sequences (NRSs) [22–24]. Plant PRVs have open circular genomes and encode a movement protein (MP), a capsid protein (CP) harboring a zinc finger motif, a protease (PR), and a reverse transcriptase with RNase H activity (RT/RH) [25]. In addition to the domains encoding these essential proteins, diverse non-standard domains or open reading frames (ORFs) have frequently been found in plant PRV genomes, the protein products of which generally play roles in vector transmission or immune suppression [26, 27]. The intergenic region (IGR) of plant PRVs, a highly diverse noncoding region containing multiple NRSs, is crucial for viral transcription, translation, and replication [25, 27]. All known PRVs encode all essential proteins and are thus autonomous PRV species during their parasitic life cycle in host cells. Limited cases of non-autonomous virus species have been previously documented. One well-known example is adeno-associated virus (*Dependoparvovirus*, a single-stranded DNA virus), which has been applied as a gene therapy vector [28]. No non-autonomous PRV species have been reported from nature to date.

In this study, we uncovered paleogenomic evidence for non-autonomous PRVs and revealed their interplay with different PRV species through an analysis of endogenous PRVs in grass family (Poaceae) genomes (S1 Table). We discovered two examples of virus–virus interactions: a possible commensal partnership between a non-autonomous PRV and an autonomous PRV species, and a possible mutualistic partnership between two functionally complementary non-autonomous PRV species. Unexpectedly, we found that the two partners in each interplaying system have frequently exchanged (>18 estimated major recombination events) their NRSs with each other via region-specific recombination to maintain partnership and coevolution. The NRS homogenization between partner viruses led by such recombination events suggests that concerted evolution has occurred in these proposed partnerships. Our results provide paleoviral insights into the genesis and adaptation of complex virus systems.

## Results

### Evidence for the existence of non-autonomous PRV species

**Fig 1:**
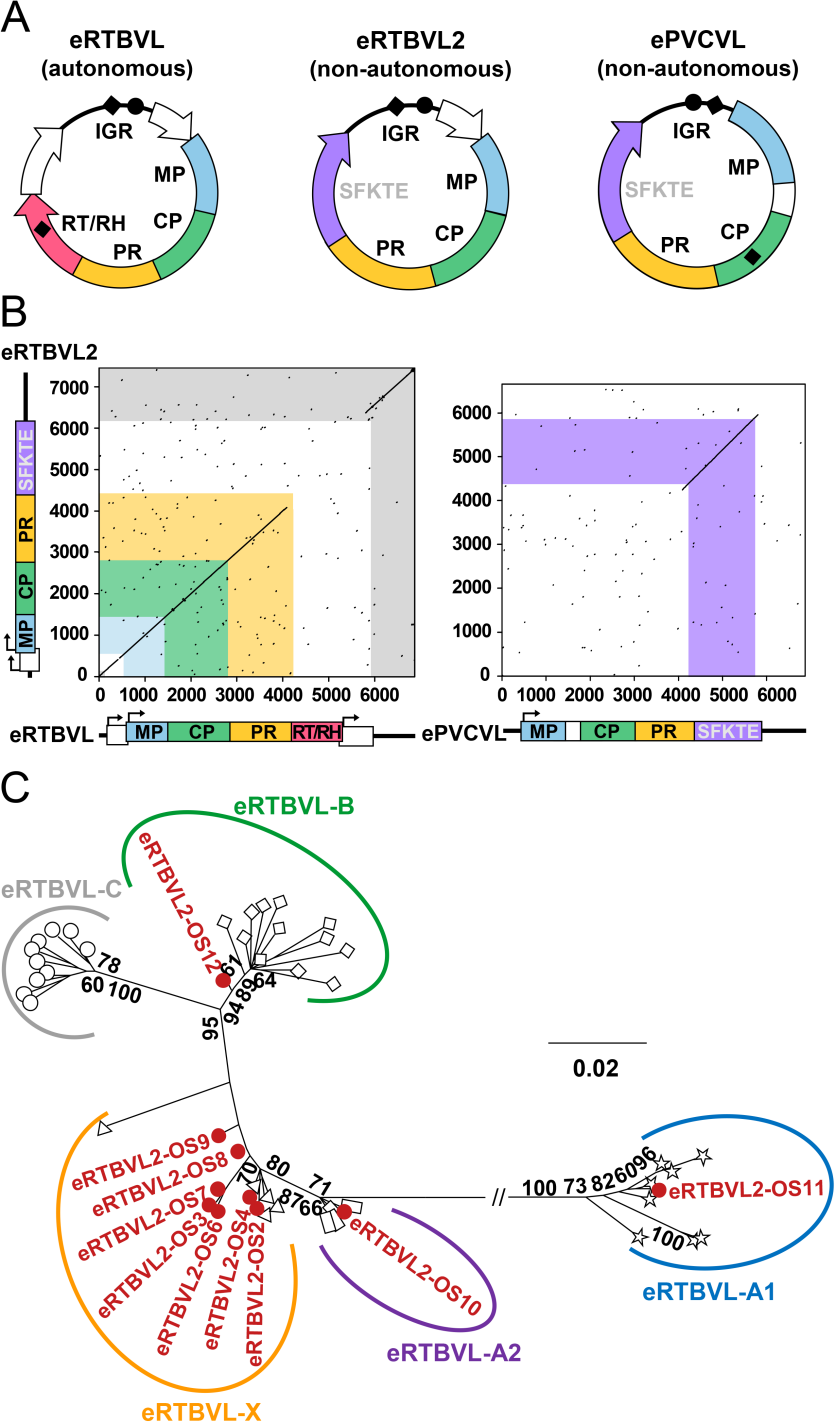
Relationship of the virus of eRTBVL2 to the viruses of eRTBVL and ePVCVL. (**A**) Ancestral virus circular genomes of eRTBVL, eRTBVL2, and ePVCVL. Open reading frames (ORFs) are represented with arrows. Predicted domains are outlined in different colors, with white used for functionally unknown regions. Viruses of eRTBVL2 and ePVCVL are defined as non-autonomous PRVs by the absence of a necessary RT/RH domain. Intergenic regions (IGRs) are represented as black curved lines. Black dots and diamonds indicate primer binding sites and polypurine tracts, respectively. The virus genome of eRTBVL was constructed according to a previous study [29], while the virus genomes of eRTBVL2 and ePVCVL were constructed from segments in the *Oryza sativa* genome (details in S2 and S3 Figs). (**B**) Dot plots of pairwise sequence comparisons of eRTBVL2 vs. eRTBVL and eRTBVL2 vs. ePVCVL. The comparisons were performed using the consensus sequence of eRTBVL (GenBank accession number BR001199.1) previously reported [30] and the consensus sequences of respective alignments of eRTBVL2 and ePVCVL sequences in the *O*. *sativa* genome (see Materials and Methods). Details of the sequence comparison using raw sequences are available in S2 Fig. Axes correspond to sequence alignment lengths. Linear virus genomic structures are shown. Rectangles with arrows indicate ORFs, and thick black lines represent IGRs. (**C**) Phylogenetic tree of IGR sequences (alignment length = 593 nt) of eRTBVL and eRTBVL2. Bootstrap support values greater than 60% based on 1,000 replicates are displayed on the unrooted maximum likelihood (ML) tree. The scale bar represents evolutionary distance in terms of substitutions per site. Sequences of various eRTBVL groups [30] are represented by white symbols, while those of eRTBVL2 are indicated by red circles. ML trees generated for other regions of eRTBVL2 are available in S6 Fig (ORF1, MP, CP, and PR domains) and S8 Fig (SFKTE domain).

**Fig 2:**
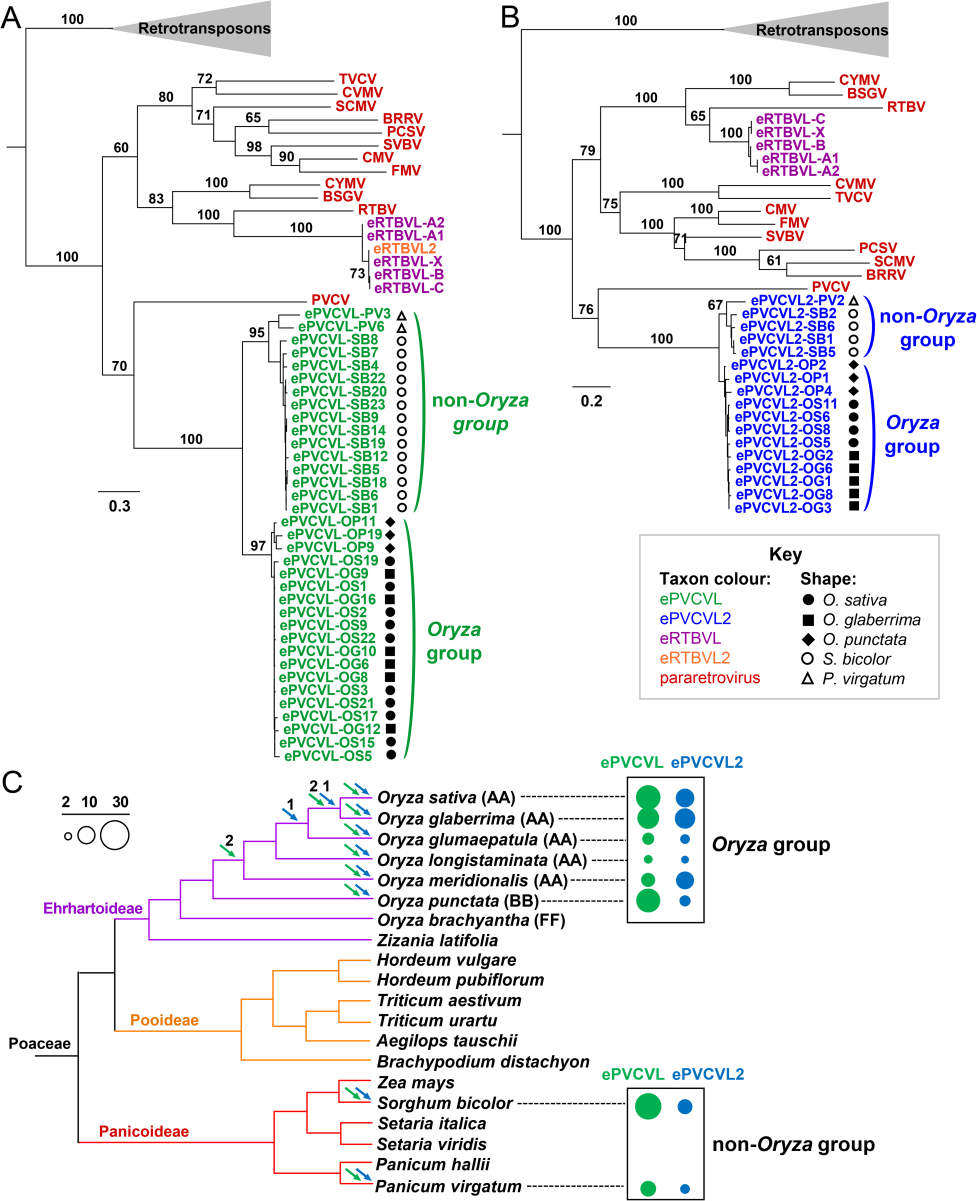
Phylogenetic relationships and distribution patterns of ePVCVL and ePVCVL2 sequences in grass genomes. (**A**) Phylogenetic relationships of ePVCVL sequences and extant PRVs based on the CP domain (alignment length = 439 amino acids). (**B**) Phylogenetic relationships of ePVCVL2 sequences and extant PRVs based on the RT/RH domain (alignment length = 415 amino acids). ML trees were constructed based on amino acid alignments. Highly truncated sequences were not included in the phylogenetic analysis. The trees are midpoint-rooted for display purposes. Bootstrap support percentages greater than 60% based on 1,000 replicates are shown above branches for major nodes. Consensus sequences of eRTBVL and eRTBVL2 were also included in the analyses. Sequences of Ty3/Gypsy LTR retrotransposons were used as outgroups. Scale bars represent genetic distances in terms of substitutions per site. Sequences of ePVCVL, ePVCVL2, eRTBVL, eRTBVL2, and extant pararetroviruses are indicated by green, blue, purple, orange, and red, respectively. The grass genome harboring each ePVCVL or ePVCVL2 sequence is represented by the indicated symbols. Detailed information on ePVCVL and ePVCVL2 sequences is available in S3 Table. CYMV, commelina yellow mottle virus; BSGV, banana streak GF virus; RTBV, rice tungro bacilliform virus; TVCV, tobacco vein clearing virus; CVMV, cassava vein mosaic virus; SCMV, soybean chlorotic mottle virus; BRRV, blueberry red ringspot virus; PCSV, peanut chlorotic streak virus; SVBV, strawberry vein banding virus; CMV, cauliflower mosaic virus; FMV, figwort mosaic virus; PVCV, petunia vein clearing virus. GenBank numbers of these sequences are available in S3 Dataset. (**C**) Endogenization and distribution of ePVCVL and ePVCVL2 in grass genomes. The phylogenetic tree of grass species was drawn according to information in references [63, 79]. Purple, orange, and red branches indicate Ehrhartoideae, Pooideae, and Panicoideae subfamily species, respectively. AA-, BB-, and FF-genome groups of the genus *Oryza* are noted in parentheses. Arrows above branches represent endogenization events in which ePVCVL (green) and ePVCVL2 (blue) segments were integrated into the corresponding grass genomes. Numerals above arrows are the number of shared ePVCVL/ePVCVL2 segments endogenized at different time points. The sizes of solid circles correspond to the number of ePVCVL (green) and ePVCVL2 (blue) segments in the different grass genomes according to the scale in the upper left hand corner.

We previously identified the first known endogenous PRV family in the genome of rice (*Oryza sativa*) [29]. This family, derived from a sister species of rice tungro bacilliform virus (RTBV)—an autonomous PRV that infects *O*. *sativa*—has been designated as endogenous RTBV-like (eRTBVL) [14, 29, 30]. In the present study, we observed domain reshuffling in at least 13 eRTBVL segments in the *O*. *sativa* genome, 7 of which formed a long cluster on chromosome 8 with segments of eRTBVL-X (the youngest group of eRTBVL [30]) (S1 Fig). These reshuffled sequences exhibited a consensus pattern among the 13 segments (S2 Fig), which suggests that the domain reshuffling must have occurred in the corresponding viral genome prior to integration. We named this reshuffled eRTBVL as endogenous RTBV-like 2 (eRTBVL2) and reconstructed its ancestral virus circular genome (Fig 1A). Instead of an RT/RH domain and a third ORF, this eRTBVL2 possessed a functionally unknown domain, henceforth referred to as the SFKTE domain (for the conserved five-residue SFKTE present in all homologous sequences) (Fig 1A and 1B). A BLAST search for the SFKTE domain sequence in the *O*. *sativa* genome identified 15 loci (*e*-value < 4.00 × 10^−44^) that have recently been annotated as endogenous PRVs similar to petunia vein clearing virus (PVCV) sequences; these PRVs are hereafter referred to as endogenous PVCV-like (ePVCVL) (Fig 2A; [18]). By aligning the regions around the identified sequences, we constructed the ancestral virus circular genome for these ePVCVL segments (Fig 1A; details in S3 Fig). The results of a detailed sequence comparison using consensus sequences of viral genomes imply a possible recombination event between the viruses of eRTBVL and ePVCVL that may have generated a recombinant virus responsible for eRTBVL2 (Fig 1B). Recombination analyses with multiple methods statistically validated this recombination event (*P* = 7.18 × 10^−309^; S2 Fig). Examination of presumed recombination breakpoints revealed no obvious sequence similarity between the parent sequences; instead, we detected a small microhomologous region at the left breakpoint (S2 Fig), which suggests an illegitimate recombination event. Three predicted essential domains (MP, CP, and PR) were confirmed by conserved motif alignment, but the RT/RH domain indispensable for replication was not detected in eRTBVL2 or ePVCVL (S2 Table and S4 Fig). Despite the absence of the RT/RH domain, the presence of multiple genomic fossils of these viruses (13 eRTBVL2 and 24 ePVCVL segments in the *O*. *sativa* genome; S2 Fig and S3 Table) suggests the success of their proliferation. We therefore propose that the viruses of eRTBVL2 and ePVCVL are non-autonomous PRV species.

### Possible commensal partnership between a non-autonomous PRV and an autonomous PRV species

To achieve replication, non-autonomous PRVs of eRTBVL2 and ePVCVL should require an autonomous partner virus or other related elements. Considering the high sequence similarity of IGRs carrying NRSs (Fig 1B; predicted NRSs in S5 Fig), we hypothesized that the virus of eRTBVL2 may depend on the protein machinery of the virus of eRTBVL (an autonomous PRV) for proliferation, similar to the case of parasitic interactions between non-autonomous and autonomous LTR retrotransposon pairs [20–22]. We thus tested the spatio-temporal likelihood of this proposed interplay. In a phylogenetic tree of IGR sequences of eRTBVL and eRTBVL2 (Fig 1C), most eRTBVL2 sequences were placed within or close to the eRTBVL-X clade, with three other eRTBVL2 sequences each falling into one of three older eRTBVL clades (-A1, -A2 and -B) [30]. Phylogenetic trees of other homologous regions (ORF1, MP, CP, and PR domains) between eRTBVL and eRTBVL2 had topologies similar to the IGR-based tree (see S6 Fig for these four ORF/domains). The results of these phylogenetic analyses suggest that recombination may have occurred between the viruses of eRTBVL and eRTBVL2 at IGRs and other homologous regions, implying their spatio-temporal coexistence. Detailed recombination analyses confirmed the contribution of the virus of eRTBVL to the recombination of the viruses of the three eRTBVL2 sequences phylogenetically close to eRTBVL-A1, -A2, and -B clades, and also supported recombination events between the viruses of eRTBVL-X and other eRTBVL2 sequences (*P* = 1.37 × 10^−9^ to 1.44 × 10^−181^; S7A Fig). We next analyzed the temporal relationship of eRTBVL2 segments based on a phylogeny of the SFKTE domain (S8 Fig). We rooted the phylogenetic tree of SFKTE amino acid sequences of eRTBVL2 and ePVCVL (S8 Fig) using the oldest ePVCVL segment, where the relative antiquity of the latter was determined by a bidirectional genome-wide orthology analysis of ePVCVL loci in *Oryza* species (see Materials and Methods and S4 and S5 Tables; PCR and Sanger sequencing validation in S9 Fig). In the generated SFKTE domain tree (S8 Fig), the eRTBVL2 segments related to the eRTBVL-X group (Fig 1C) were the latest branching sequences, whereas the three eRTBVL2 segments related to eRTBVL-A1, -A2, and -B groups (Fig 1C) branched earlier (S8 Fig). Because the eRTBVL-X group is the youngest eRTBVL group and eRTBVL-A1, -A2, and -B groups are older [30], the SFKTE phylogeny indicates that the evolution of the virus of eRTBVL2 is temporally consistent with that of eRTBVL. Taken together, these results strongly support the coexistence and coevolution of the viruses of eRTBVL2 and eRTBVL and provide evidence for a possible partnership between the two viruses during mixed infection. The virus of eRTBVL2 did not seem to be a parasite on the virus of eRTBVL, because we observed no higher magnitude of proliferation in the former relative to the latter (Fig 1C and S6 Fig). Taking into account the observation that the replication dependence of the virus of eRTBVL2 on the virus of eRTBVL had no recognizable deleterious effect on the latter, we suggest a possible commensal partnership between the viruses of eRTBVL2 and eRTBVL.

### Possible mutualistic partnership between complementary non-autonomous PRV species

**Fig 3:**
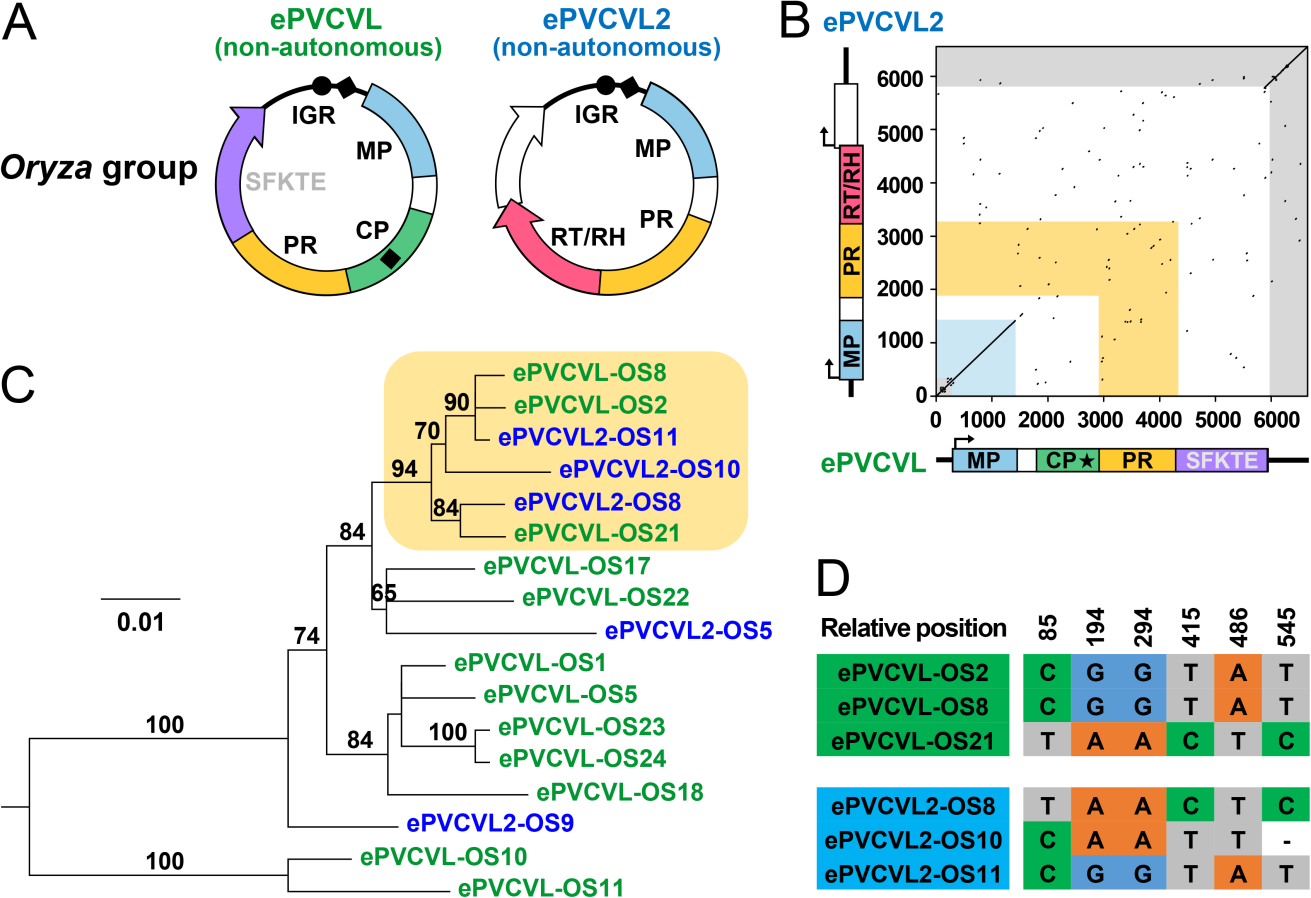
Viral genome comparison and phylogenetic analysis of IGR sequences between the viruses of ePVCVL and ePVCVL2 *Oryza* groups. (**A**) Ancestral virus circular genomes of ePVCVL and ePVCV2 *Oryza* groups. ORFs are represented with arrows. Predicted domains are outlined in different colors, with white used for functionally unknown regions. Viruses of ePVCVL2 are defined as non-autonomous PRVs by the absence of a necessary CP domain. IGRs are represented as black curved lines. Black dots and diamonds indicate primer binding sites and polypurine tracts, respectively. Details on virus genome construction are provided in S3 Fig. (**B**) Dot plots of pairwise sequence comparisons of ePVCVL vs. ePVCVL2 *Oryza* groups using consensus sequences of the respective alignments of ePVCVL and ePVCVL2 sequences in the *O*. *sativa* genome. Axes correspond to sequence alignment lengths. Linear virus genomic structures are shown. Rectangles with arrows indicate ORFs, and thick black lines represent IGRs. Black stars represent the zinc finger motif in the CP domain. (**C**) Phylogenetic relationships of IGR sequences (alignment length = 538 nt) of ePVCVL and ePVCVL2 in the *O*. *sativa* genome. The ML tree was rooted using the oldest identified ePVCVL segment (Fig 2C, S4 and S5 Tables). ePVCVL and ePVCVL2 sequences in the tree are indicated by green and blue, respectively. Bootstrap support values greater than 60% based on 1,000 replicates are shown as percentages at branches. The scale bar represents evolutionary distance in terms of nucleotide substitutions per site. The tree of IGR sequences of ePVCVL and ePVCVL2 in other *Oryza* genomes is shown in S10 Fig. (**D**) Single nucleotide polymorphisms shared by ePVCVL and ePVCVL2 sequences in the orange-highlighted clade of the tree. Relative positions in the alignment are shown, and gaps in the alignment are indicated by hyphens.

Although our search for the autonomous partner of the virus of ePVCVL revealed no such candidate in the genomes of *O*. *sativa* or other *Oryza* species, we noticed another endogenous PVCV-like family (hereafter ePVCVL2) showing defective structures (Fig 2B and 2C; [18]). We successfully reconstructed the ancestral virus circular genome of ePVCVL2; this ancestral genome possessed MP, PR, and RT/RH domains but the CP domain was absent (Fig 3A; details in S2 Table, S3 and S4 Figs). The composition of this genome suggests that the virus of ePVCVL2 is structurally and functionally complementary to the virus of ePVCVL. Given the existence of the naturally defective genome as well as multiple fossils of the virus of ePVCVL2 (11 segments in the *O*. *sativa* genome; S3 Table), we suggest that this virus is another non-autonomous PRV species. Detailed comparison of ePVCVL and ePVCVL2 consensus sequences revealed a high degree of local similarity between their IGRs as well as their MP domains (97.2% nucleotide identity: 99.3% for IGR and 95.3% for MP) (Fig 3B). Given that IGR sequence identities between eRTBVL groups (intraspecies level) ranged from 72.6% to 92.8%, this interspecies similarity of IGRs is exceptionally high. Both ePVCVL and ePVCVL2 encode a PR domain, but the nucleotide sequence of this region was very dissimilar between these two types of endogenous PRVs (Fig 3B). This dissimilarity of PR domains, extraordinarily high IGR sequence similarity (identical NRSs between IGRs; predicted NRSs in S5 Fig), and observed functional complementarity between the viruses of ePVCVL and ePVCVL2 all suggest a possible mutualistic partnership in which the two viruses mutually compensate to facilitate proliferation.

To confirm the proposed partnership, we performed a bidirectional genome-wide orthology analysis of ePVCVL2 loci in *Oryza* genomes (the same analysis of ePVCVL loci mentioned above). This analysis revealed that ePVCVL and ePVCVL2 segments are species-specific, except for four shared ePVCVL loci and two shared ePVCVL2 loci, and coexist in each analyzed *Oryza* genome (Fig 2C; details in S9 Fig, S4 and S5 Tables), thereby supporting the coexistence of the viruses of ePVCVL and ePVCVL2 during host divergence. No major ePVCVL cluster related to a major ePVCVL2 cluster was present in the phylogenetic tree of ePVCVL and ePVCVL2 IGR sequences in the *O*. *sativa* genome (Fig 3C). On the contrary, three ePVCVL IGR sequences clustered with three ePVCVL2 IGR sequences in a strongly supported clade (Fig 3C). To confirm this finding, we examined single nucleotide polymorphisms (SNPs) among the six IGR sequences, which revealed six SNP sites shared by the IGRs of ePVCVL and ePVCVL2 (Fig 3D). We further carried out recombination analyses on these ePVCVL and ePVCVL2 sequences, which resulted in the identification of significant recombination events between the IGRs of the viruses of ePVCVL and ePVCVL2 (*P* = 1.28 × 10^−8^ to 2.90 × 10^−23^; S7B Fig). When we extended our phylogenetic analysis of IGR sequences to segments in other *Oryza* genomes, we also found that the IGR sequences of ePVCVL and ePVCVL2 clustered together (S10 Fig). The recombination of IGR sequences between the viruses of ePVCVL and ePVCVL2, implied by the phylogenetic analysis, was likewise confirmed by recombination analyses of ePVCVL and ePVCVL2 sequences in these *Oryza* genomes (*P* = 4.06 × 10^−4^ to 6.87 × 10^−23^; S7C Fig). Taken together, these data thus provide strong evidence that two non-autonomous PRVs in a possible mutualistic partnership have recombined their IGR sequences to continue their coevolution during mixed infection.

### Another example of interplay between different non-autonomous PRV species

**Fig 4:**
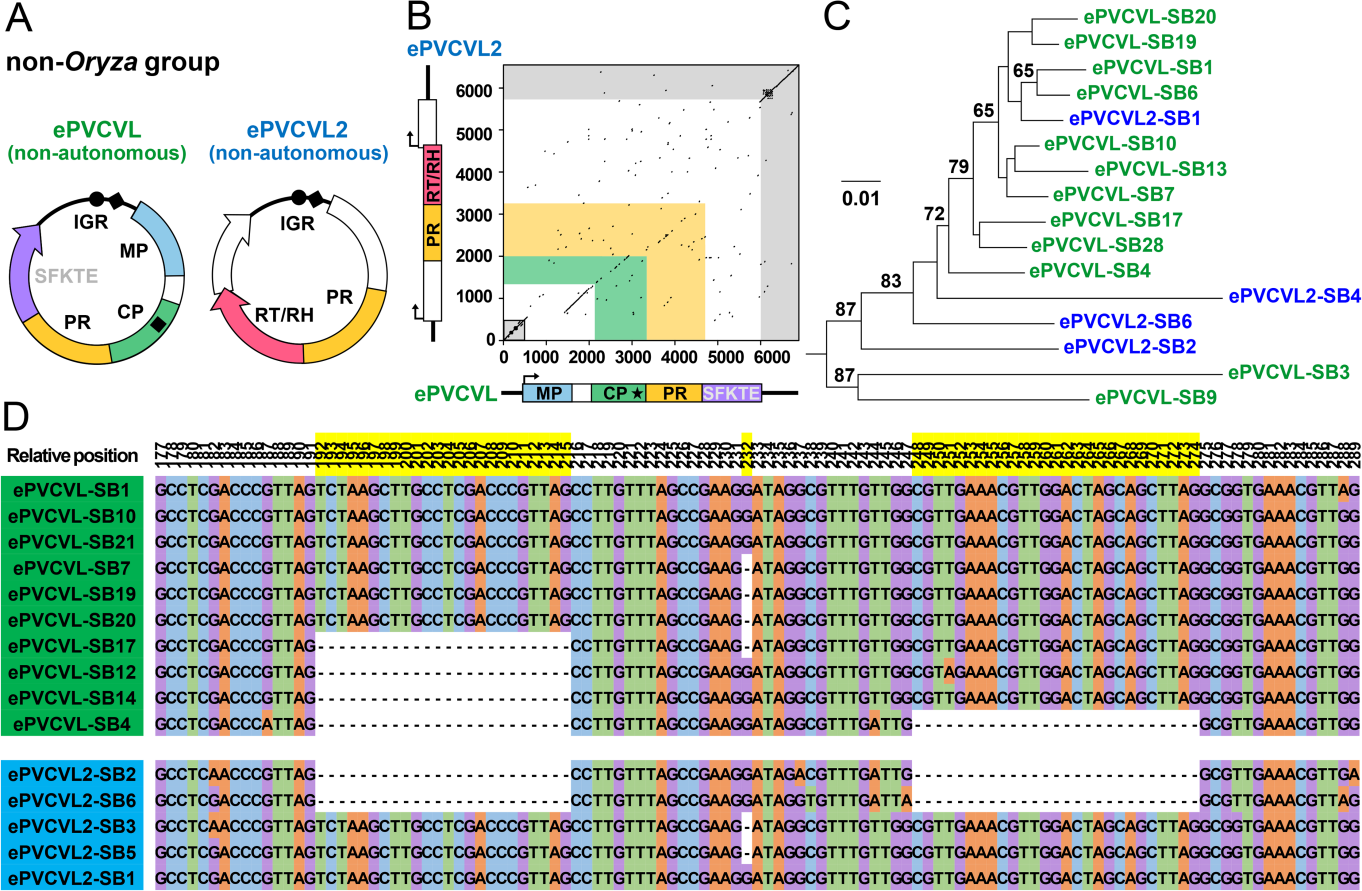
Viral genome comparison and phylogenetic analysis of IGR sequences between the viruses of ePVCVL and ePVCVL2 non-*Oryza* groups. (**A**) Ancestral virus circular genomes of ePVCVL and ePVCV2 non-*Oryza* groups. ORFs are represented with arrows. Predicted domains are outlined in different colors, with white used for functionally unknown regions. IGRs are represented as black curved lines. Black dots and diamonds indicate primer binding sites and polypurine tracts, respectively. Details on virus genome construction are provided in S3 Fig. (**B**) Dot plots of pairwise sequence comparisons of ePVCVL vs. ePVCVL2 non-*Oryza* groups using consensus sequences of respective alignments of ePVCVL and ePVCVL2 sequences in the *Sorghum bicolor* genome. Axes correspond to sequence alignment lengths. Linear virus genomic structures are shown. Rectangles with arrows indicate ORFs, and thick black lines represent IGRs. Black stars represent the zinc finger motif in the CP domain. (**C**) Phylogenetic relationships of IGR sequences (alignment length = 589 nt) of ePVCVL and ePVCVL2 in the *S*. *bicolor* genome. The ML tree was midpoint-rooted for display purposes. ePVCVL and ePVCVL2 sequences in the tree are indicated by green and blue, respectively. Bootstrap support values greater than 60% based on 1,000 replicates are shown as percentages at branches. The scale bar represents evolutionary distance in terms of nucleotide substitutions per site. (**D**) Examples of virus-derived small indel variations in IGR sequences of ePVCVL and ePVCVL2 in the *S*. *bicolor* genome. The relative positions of variations in the alignment are highlighted in yellow (absolute positions in viral genomes = 153–289).

By searching for homologous sequences of eRTBVL2, ePVCVL, and ePVCVL2 and reexamining reported endogenous PRVs in non-*Oryza* grass genomes [18], we found both ePVCVL and ePVCVL2 homologous sequences coexisting in the genomes of sorghum (*Sorghum bicolor*) and switchgrass (*Panicum virgatum*) (Fig 2 and S3 Table). These sequences formed a phylogenetic sister group (non-*Oryza* group) to either ePVCVL or ePVCVL2 segments of analyzed *Oryza* genomes (*Oryza* group) (Fig 2A and 2B). We constructed two ancestral virus circular genomes for these sequences (Fig 4A; details in S3 Fig). One was structurally equivalent to *Oryza* group ePVCVLs, that is, the RT/RH domain was absent. The other genome resembled *Oryza* group ePVCVL2s, but lacked both MP and CP domains (this genome contained a region slightly resembling the CP domain but without an essential zinc finger motif) (Fig 4A and 4B; details in S2 Table, S3 and S4 Figs).

IGR sequences of ePVCVL and ePVCVL2 non-*Oryza* groups shared extremely high nucleotide identities (97.1%; Fig 4B), whereas IGR sequence similarities between ePVCVL *Oryza* and non-*Oryza* groups and between ePVCVL2 *Oryza* and non-*Oryza* groups were low (43.6% and 44.6% nucleotide identities, respectively; S11 Fig). In a phylogenetic tree based on sequences from the *S*. *bicolor* genome, IGR sequences of non-*Oryza* ePVCVL and ePVCVL2 groups were mixed together (Fig 4C). (The number of IGR sequences in the *P*. *virgatum* genome was too limited for phylogenetic analysis). We also performed recombination analyses on these sequences in the *S*. *bicolor* genome, which resulted in the detection of significant recombination events occurring between the IGRs of the viruses of ePVCVL and ePVCVL2 non-*Oryza* sequences (*P* = 6.30 × 10^−10^ to 1.23 × 10^−22^; S7D Fig). A close examination of the *S*. *bicolor* sequences revealed that virus-derived small insertion/deletion (indel) variations in IGRs were shared between partial non-*Oryza* ePVCVL and ePVCVL2 segments (Fig 4D). The presence of these indels is direct evidence that IGRs have frequently been recombined between the virus genomes of ePVCVL and ePVCVL2. Taking all of these results into consideration, we conclude that non-autonomous PRVs have adapted to a long-term partnership via IGR homogenization mediated by frequent recombination, leading to concerted evolution of NRSs.

## Discussion

**Fig 5:**
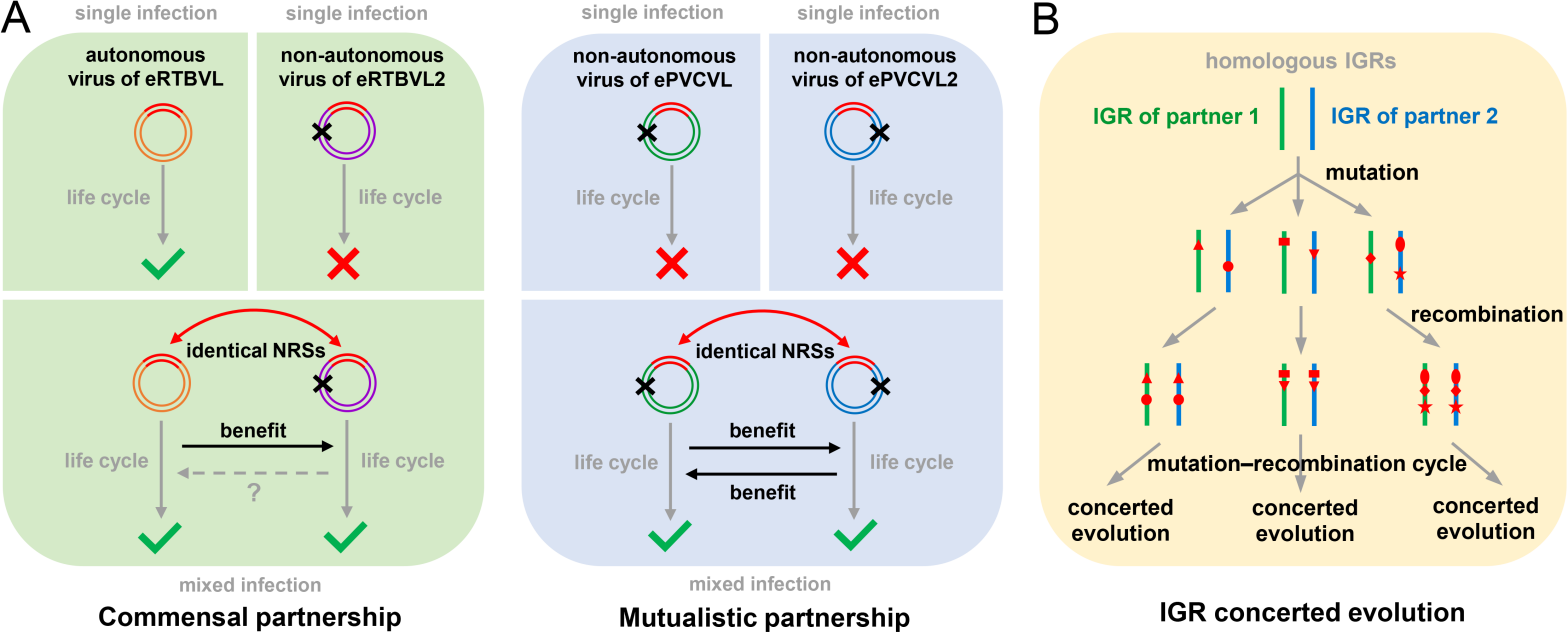
Proposed models for interplay between non-autonomous PRVs and their partners. (**A**) Possible partnerships between a non-autonomous PRV and an autonomous PRV species and between different non-autonomous PRV species. Different-colored ring sections illustrate different viral genomes (orange, purple, green, and blue for eRTBVL, eRTBVL2, ePVCVL, and ePVCVL2, respectively). Red sections represent IGR sequences, with superimposed black crosses indicating structural defects. Green check marks and red crosses respectively indicate successes and failures of viral life cycles in hosts. Double-headed red arrows refer to the identical NRSs in IGRs between two partner viruses, while black arrows represent conferral of benefits on a partner virus during mixed infection. Uncertainty with respect to the latter is indicated by a dashed gray arrow. (**B**) Concerted evolution of IGR sequences between partner viruses. Green and blue lines represent the homologous IGR sequences of two partner viruses. Different red symbols indicate various mutations.

The discovery and analysis of various EVEs in eukaryotic genomes has contributed to our understanding of viral origin and evolution as well as long-term interactions between viruses and hosts [3, 31–33]. Endogenous PRVs in plant genomes have been frequently reported [5, 18, 34], and extreme cases of endogenous PRV reactivation under certain conditions, such as in endogenous banana streak virus, have been well documented [35–38]. Using grass endogenous PRVs as ancient DNA records of viruses, we performed paleogenomic analyses of PRVs to explore their long-term virus–virus interactions. In contrast to all previously known PRVs, which are autonomous, three non-autonomous PRV species were identified in this study, namely, the viruses of eRTBVL2, ePVCVL, and ePVCVL2. Our examination of ePVCVL and ePVCVL2 sequences, which were first described by Geering *et al*. [18], revealed the adaptation strategies of their corresponding non-autonomous viruses. We have proposed two adaptation strategies used by non-autonomous PRVs: a possible commensal partnership with autonomous PRVs and a possible mutualistic partnership with other non-autonomous PRVs (summarized in Fig 5A). These proposed partnerships have been enabled by the existence of shared common NRSs in their IGRs. We have also demonstrated the evolutionary dynamics of these partnerships: frequent recombination of IGRs (>18 estimated major events; see below) between two partners leading to NRS homogenization between different PRV species during host divergence. This concerted evolution of NRSs is responsible for the maintenance of such partnerships and has driven the coevolution of interacting viruses.

The consensus NRSs of two partner viruses would be expected to recruit the same virus-encoded proteins and host factors to complete their life cycles in hosts. In the possible commensal partnership suggested by this study (Fig 5A), the non-autonomous virus of eRTBVL2 should benefit from sharing the RT/RH protein of the autonomous virus of eRTBVL. With respect to the SFKTE domain of the virus of eRTBVL2, neither the RT-like motif nor its degenerate residues could be distinguished in this domain by amino acid alignment with all known types of RT-like domains (S4 Fig and S1 Dataset) or by using HHpred, a sensitive detection method based on profile hidden Markov models (S2 Table; see Materials and Methods) [39]. Although the possibility cannot be completely excluded and future biochemical verification is needed, the likelihood of RT activity in SFKTE proteins is very low. In fact, plant PRV genomes usually possess various additional non-standard domains or ORFs that often play a role in vector transmission or immune suppression [26, 27]. SFKTE proteins may have functions similar to those of well-known additional PRV proteins, such as interaction with insect vector proteins or host antiviral factors [26, 27]. Although not necessary for its replication, the virus of eRTBVL may also benefit, to some extent, from such a function of SFKTE proteins encoded by the virus of eRTBVL2 during mixed infection. Consequently, an alternative relationship may exist between the two viruses: a mutualistic partnership. In the possible mutualistic partnership suggested for the viruses of ePVCVL and ePVCVL2 (Fig 5A), the two non-autonomous viruses benefit from each other via functional complementary. The RT/RH protein from the virus of ePVCVL2 reverse transcribes its own pregenomic RNA as well as that of the virus of ePVCVL, while the CP protein from the virus of ePVCVL assembles its own viral particles as well as those of the virus of ePVCVL2. Products from additional domains/ORFs of these two viruses (the SFKTE domain of the virus of ePVCVL and ORF2 of the virus of ePVCVL2) may also contribute to the putative mutualistic partnership. In the case of the non-*Oryza* group, the MP protein from the virus of ePVCVL is responsible not only for its own cell-to-cell movement, but also for that of the virus of ePVCVL2; at the same time, the region in the virus of ePVCVL2 slightly similar to the CP domain but lacking a zinc finger motif may encode defective CP proteins (i.e., those lacking viral DNA binding activity because of missing zinc finger motifs) to bind host antiviral proteins to disable viral-CP-binding activities. This system of two interplaying viruses is reminiscent of extant complex viruses possessing multiple polynucleotide sequences, which suggests that functional complementarity and co-regulation may have contributed to the origin of multipartite viruses.

The interspecies recombination event that generated the virus of eRTBVL2 (Fig 1B and S2 Fig) occurred between the viruses of eRTBVL (*Tungrovirus*-related species [29]) and ePVCVL (*Petuvirus*-related species; Fig 2A), which belong to different genera and possess distinct genomic structures with very weak sequence similarities. The presence of reshuffled domain combinations in the viral genome of eRTBVL2 relative to the virus of eRTBVL (Fig 1A and 1B) supports the theory of modular evolution that has been considered to be applicable to all known virus types [40, 41]. Putative interspecies recombination events have frequently been reported in viruses [42–46]. We propose that interspecies recombination is one of the mechanisms driving viral modular evolution. We particularly note that the frequent exchange of IGRs revealed in this study implies that modular evolution applies not only to coding domains, but also possibly to NRSs. Other studies have observed that recombination between endogenous and exogenous retroviruses has occasionally occurred and produced recombinant viruses [47–52]. This recombination may occur when exogenous and endogenous retroviral RNAs are coexpressed in host cells [47]. Recombination between endogenous and exogenous PRVs has not been reported to date [12]. Although in our study we also found no evidence to support the origin of any non-autonomous PRVs from such recombination, consideration of the evolutionary influence of this type of occasional albeit hypothetical recombination event is still of interest.

Concerted evolution has been widely observed to accompany the sequence homogenization process of some duplicated genes or elements in prokaryotic and eukaryotic genomes; one notable example is the sequence homogenization of ribosomal DNA repeats within a species [53, 54]. Concerted evolution has also been reported in nanoviruses, which are single-stranded DNA viruses [55, 56]. In our study, concerted evolution was observed during the homogenization of IGRs between a pair of partner viruses. IGRs are noncoding and highly divergent across PRV genomes; for example, IGRs of RTBV and PVCV respectively share less than 44.4% and 35.1% nucleotide identities with those of other PRVs (NCBI genome database). Nevertheless, the overall set of IGRs (and neighboring regions) between the two partner viruses in this study displayed an extraordinarily high sequence similarity (Figs 1B, 3B and 4B). This finding suggests that recombination, rather than mutation selection, is the main contributor to IGR homogenization between partner viruses. The results of our detailed phylogenetic and recombination analyses support the idea that persistent recombinations have driven this IGR concerted evolution (Figs 1C, 3C and 4C; S7 and S10 Figs). When we generated consensus sequences for eRTBVL2, ePVCVL, and ePVCVL2, we found a consensus pattern for each recombination breakpoint (Figs 1B, 3B and 4B, S2 and S3 Figs). This discovery suggests that these recombinations took place between homologous localized regions of two partner viruses; in other words, the recombinations were region-specific [23]. We propose the following model to explain the process of concerted evolution of IGR sequences (Fig 5B). Once illegitimate recombination produced identical (or highly similar) IGR sequences between the viruses of eRTBVL and eRTBVL2, mutations accumulated in these IGRs over time; however, region-specific recombination within homologous IGRs (and neighboring regions) of the two viruses exchanged these mutations between virus populations during mixed infection, with subsequent recombination within a viral population able to further spread the exchanged mutations. The constant repetition of this mutation–recombination cycle caused the two viruses in the putative partnership to maintain highly similar IGRs. As one of the two partner viruses diverged into a new lineage during evolution, the other coevolved via region-specific recombination between their homologous regions; this resulted in different viruses of eRTBVL2 possessing different IGRs that were highly similar to those of each of the viral lineages of eRTBVL groups (Fig 1C). Likewise, the constant repetition of this mutation–recombination cycle during the evolution of the viruses of ePVCVL and ePVCVL2 caused each partner of the virus pair infecting the same grass species to always maintain highly similar IGRs, even as the viruses of ePVCVL/ePVCVL2 diverged into distinct lineages infecting different host species in different habitats (Figs 3C and 4C, and S10 Fig). Consequently, divergent evolution occurred in each of the four studied virus species, whereas concerted evolution took place between the IGRs of each pair of partner viruses (Fig 5B).

Although precise quantification of the recombination frequency in these viral partnerships appears to be difficult, we tried to estimate the number of major recombination events between IGRs of partner viruses based on phylogeny. Phylogenetic clustering of eRTBVL2 IGRs with those of each of four eRTBVL groups (Fig 1C) suggested the occurrence of more than four major recombination events. Similarly, a total of 10 major recombination events were suggested by phylogenetic analyses of ePVCVL and ePVCVL2 IGRs (Figs 3C and 4C, and S10 Fig). In regards to the remaining grass genomes, which were not phylogenetically analyzed because of the high truncation and limited number of sequences, the independent endogenization and IGR concerted evolution of ePVCVL and ePVCVL2 in each genome imply that more than one major recombination event has taken place in each genome (a total of four) (Fig 2C and S3 Table). We consequently detected more than 18 independent major recombination events, which supports the idea that partner viruses have frequently recombined IGRs with each other to maintain partnership and coevolution. Although recombination has probably been much more frequent than we have estimated, these major events have had significant impacts on viral phylogeny during long-term evolution.

Similar to the recombination of retroviruses, PRVs such as cauliflower mosaic virus (CaMV) have been thought to recombine mostly through intermolecular template switching during reverse transcription in the host cytoplasm [23, 57, 58]. In our study, however, locational patterns of viral strand discontinuities (primer binding sites and polypurine tracts) did not correspond well to patterns of sequence similarity between viral genomes (Figs 1, 3 and 4, S2 and S3 Figs). When present in the host nucleus, PRV DNA is organized into minichromosomes [27], and indirect evidence exists that CaMV recombinations sometimes take place between viral minichromosomes [59, 60]. Consequently, the region-specific recombinations identified in this study may have occurred mainly through homologous recombination between local homologous regions of viral minichromosomes with the help of host recombination machinery. One homologous recombination mechanism, gene conversion, has been suggested to be responsible for the concerted evolution of ribosomal DNA and other genes [53, 61, 62].

Our study has provided paleogenomic evidence for non-autonomous PRVs as well as their adaptation. Considering the abundance of diverse EVEs harbored in eukaryotic genomes and the rapid accumulation of genomic data [3], many EVEs derived from previously unknown unusual virus types may still await discovery and analysis. At the same time, plentiful remnants of ancient virus–virus interactions may have been recorded in host genomes; our study has revealed one such paleovirological case of interplay between viral NRSs. One important future research focus should be evaluation of the prevalence and dynamics of NRS interactions between viral pathogens in mixed infections in plants and humans or within a viral population, as these may have significant impacts on viral evolution and pathology.

## Materials and methods

### Genomic data mining and virus genome reconstruction

Whole-genome sequences of 20 grass species were downloaded mainly from the Gramene database [63] (detailed data sources in S1 Table). To identify endogenous PRVs, we first performed a BLASTn search (with default settings) using the BLAST+ 2.2.27 utility and previously reported sequences [64]. The hit sites (*e*-values < 1 × 10^−10^ and lengths >100 bp) along with their 5,000-bp upstream and downstream sequences were retrieved and assembled into consensus sequences (the nucleotide with the highest frequency at each position in the alignment was selected) using the Vector NTI Advance 11.5 toolkit (Invitrogen). A second round of BLASTn searching and a BLASTp search were then performed using these consensus sequences and their translated amino acid sequences, respectively. Only hit sequences longer than 100 bp were retained. Each translated protein sequence was subjected to the HHpred server [39], with all standard HHM databases (as of 3 May 2014) chosen for homologous domain detection (using default parameters). To check unidentified domains/ORFs, their amino acid sequences were resubmitted to the HHpred server and also subjected to BLASTp and tBLASTn searches against NCBI databases. Identified domains were confirmed by conserved motif alignment. Coordinates of eRTBVL2, ePVCVL, and ePVCVL2 sequences and their genes/regions in grass genomes are available in S2 Dataset (BED format). Dot plots were generated using the EMBOSS package (word size = 10; threshold = 45) [65].

### Phylogenetic analyses

Nucleotide sequences of each dataset were aligned in ClustalW [66] followed by manual editing. After being translated from the aligned nucleotide sequences, amino acid sequences of each dataset were realigned using MUSCLE [67] followed by manual editing. Highly truncated sequences (generally shorter than 80% of the entire region) and ambiguous regions were removed from the final alignments. Best-fitting substitution models were determined for each aligned dataset according to the Akaike information criterion calculated using jModelTest version 2.1.4 [68] or ProtTest version 3.2 [69]. For eRTBVL2 datasets comprising IGR (nucleotide positions 6063–6704 of the consensus genome), MP (486–1853), CP (1854–2845), PR (2831–4090), and ORFx (48–485) sequences, the best-fitting models were HKY+G, TrN+G, GTR+G, TrN+I+G, and TrN+G, respectively, with JTT+I+F chosen for the SFKTE sequences corresponding to amino acid positions 1220–1741 of the ORF2 protein sequence. Models VT+F+G and LG+I+F+G were respectively selected for the ePVCVL CP dataset (amino acid positions 709–996/722–1010 of the protein sequence of *Oryza*/non-*Oryza* groups) and the ePVCVL2 RT/RH dataset (amino acid positions 1017–1414/945–1342 of the ORF1 protein sequence of *Oryza*/non-*Oryza* groups). Models HKY+G, GTR+G, and HKY+G were respectively chosen for the IGR datasets of ePVCVL and ePVCVL2 of *O*. *sativa*, genus *Oryza*, and *S*. *bicolor* genomes (nucleotide positions 5878–6415/5786–6323, 5878–6611/5786–6519, and 6008–6659/5691–6317 of the consensus genomes of ePVCVL/ePVCVL2, respectively). Maximum-likelihood (ML) phylogenetic analyses were performed in PhyML version 3.0 [70] or MEGA version 6.06 (only for Fig 1C and S6 Fig for display purposes) [71]. Branch support in all trees was calculated using 1,000 bootstrap replicates. The tree for the SFKTE domain of eRTBVL2 and ePVCVL segments was rooted using the oldest ePVCVL segment as determined by orthology analysis of ePVCVL loci in *Oryza* species (see below). ePVCVL was assumed to be older than eRTBVL2, as eRTBVL2 only exists in a subspecies of *O*. *sativa*, whereas ePVCVL is present in all *O*. *sativa* subspecies (see S8 Fig). All sequence alignments for phylogenetic analyses are available in S3 Dataset.

### Recombination analyses

Sequences suggested as having a high probability of recombination according to the phylogenetic analyses and sequence alignments were subjected to recombination analyses using RDP version 4.72 [72]. We used six different methods (RDP [73], GENECONV [74], BootScan [75], MaxChi [76], Chimaera [77], and SiScan [78]) in this program to identify potential recombination events and perform statistical tests. Sequence alignments for the recombination analyses were generally extracted from the alignment datasets of phylogenetic analyses. In the case where no suitable phylogenetic dataset was available, sequence alignments used for recombination analyses were made in MUSCLE [67] followed by manual editing. Default parameters were used for each method, except that the reference sequence parameter of the RDP method, in accordance with the RDP manual, was adjusted to “internal references only” when many closely related sequences existed in the alignment [72]. For each method, *P* < 0.005 was used as a threshold value for possible recombination events. Only the recombination events independently detected by more than three methods with statistical significance were considered reliable, and the best *P* value for each event was chosen. These recombination events were checked and displayed in BootScan plots (window size = 300 nt; step size = 10 nt) using the RDP program. All alignments used for recombination analyses are available in S4 Dataset.

### Genome-wide orthology analysis

If an ePVCVL/ePVCVL2 segment in an *Oryza* genome was located next to or near another ePVCVL or ePVCVL2 segment (i.e., less than 5 kb away on the same chromosome or scaffold), the two (or more) segments were generally considered to be one locus for the analysis. The left and right 5-kb flanking sequences of each locus of ePVCVL and ePVCVL2 in the *O*. *sativa* genome were first mapped onto five other *Oryza* genomes (*O*. *glaberrima*, *O*. *glumaepatula*, *O*. *longistaminata*, *O*. *meridionalis*, and *O*. *punctata*) using BLASTn. The mapping results were rechecked using genome collinearity data (genome-wide alignments between *Oryza* genomes) obtained from the Gramene database [63]. Both 5-kb flanking sequences of each locus of ePVCVL and ePVCVL2 in the five above-mentioned *Oryza* genomes were next mapped onto the *O*. *sativa* genome and rechecked in the same manner. Some flanking sequences in *O*. *glumaepatula* and *O*. *meridionalis* genomes contained many uncharacterized (‘N’) bases; the examined length of these flanking sequences was therefore extended to 15 kb.

### Plant materials, polymerase chain reaction (PCR) amplification, and sequencing

Genomic PCR and Sanger sequencing were used to confirm orthologous loci of ePVCVL and ePVCVL2. Loci shared among *Oryza* species were examined; in addition, representative *O*. *sativa*-specific ePVCVL and ePVCVL2 loci were selected and analyzed. Wild and cultivated rice plants (accession numbers in S9 Fig) were grown in a greenhouse at Hokkaido University, Sapporo, Japan. Total DNA was extracted from leaf samples using cetyltrimethylammonium bromide extraction buffer. DNA concentrations were all diluted to the same order of magnitude. PCR amplifications were performed using Ex *Taq* or LA *Taq* polymerase (Takara) on a PTC-200 thermal cycling system (GMI). PCR products were resolved on a 1–2% agarose gel, stained with ethidium bromide, and viewed using an AE-6933FXES Printgraph system (ATTO). Sanger sequencing was performed on an ABI 3730 DNA Analyzer (Applied Biosystems) using a BigDye Terminator v3.1 cycle sequencing kit (Applied Biosystems) according to the manufacturer’s protocol. Information on the primers used in this study is provided in S6 Table.

### DNA sequences

All relevant data are within the paper and its Supporting Information files except for the assembled sequences of non-autonomous PRVs, which are available from DDBJ database under accession numbers BR001403–BR001407.

## Supporting information

S1 Fig Clustering of eRTBVL2 and eRTBVL-X sequences on chromosome 8 of the *Oryza sativa* genome. Blue, light green, and dark green indicate eRTBVL2, eRTBVL-X, and ambiguous (eRTBVL2 or eRTBVL-X) sequences, respectively. The structures of eRTBVL2 and eRTBVL-X sequences are marked with red fonts and their lengths are drawn roughly to scale. These eRTBVL2 segments were formerly undistinguished from eRTBVL-X segments in the eRTBVL-X cluster [30].
(PDF)

S2 Fig Recombinational origin of the virus of eRTBVL2. The results of recombination analyses of the viral genomes of eRTBVL, eRTBVL2, and ePVCVL are shown at the top. The alignment for the analyses was made using viral consensus sequences constructed from the *O*. *sativa* genome (eRTBVL consensus sequence GenBank accession number: BR001199.1). The table in the upper left hand corner summarizes the recombination events detected by different methods. NS, not significant. Recombination events were checked using the BootScan plot shown on the right. In the plot, the vertical axis indicates the supporting percentage of pair-wise distance measurements based on 100 bootstrap replicates, and the horizontal axis indicates the relative position in the alignment. The bar at the top of the plot indicates informative sites in the alignment. The alignment of eRTBVL, eRTBVL2, and ePVCVL sequences at possible recombination breakpoints of their viral genomes is shown at the bottom. Segment names are shown to the left of the alignment, and segments with large deletions are highlighted in gray. Selected regions of eRTBVL2 raw (non-consensus) sequences are displayed and aligned to the corresponding regions of eRTBVL and ePVCVL consensus sequences. Regions that are highly similar between eRTBVL and eRTBVL2 sequences are indicated by red lines, while those between ePVCVL and eRTBVL2 sequences are framed in black. Suggested recombination breakpoints are marked by arrows. The microhomologous region is indicated by a yellow line. Forward slashes and hyphens represent sequence omissions and aligned gaps, respectively.
(PDF)

S3 Fig Reconstruction of the ancestral virus circular genomes of ePVCVL and ePVCVL2 sequences from different grass genomes. Viral genomes were reconstructed from the genomes of *O*. *sativa*, *O*. *glaberrima*, *O*. *punctata*, and *Sorghum bicolor*, for which multiple long ePVCVL and ePVCVL2 segments were available for reconstruction. Open reading frames (ORFs) are represented by arrows, and predicted domains are outlined in different colors (white for functionally unknown regions). Intergenic regions (IGRs) are represented by black curved lines. Black stars represent the zinc finger motif in the CP domain, and black dots and diamonds indicate primer binding sites and polypurine tracts, respectively. Segment sequences used in reconstructions are represented by blue curved lines in the outer portions of the viral genomic structures and are matched to the corresponding positions of viral genomes (detailed segment information is available in S3 Table).
(PDF)

S4 Fig Motif alignments for the four essential domains of grass endogenous PRVs and various extant PRVs. Portions of sequence alignments are shown for conserved motifs in MP (**A**), CP (**B**), PR (**C**), and RT/RH (**D**) domains. Each conserved motif is indicated by a red line. Slashes represent sequence omissions.
(PDF)

S5 Fig Predicted elements of noncoding regulatory sequences in IGRs. The putative short ORF (blue box), stem-loop structure (green line), promoter (orange semi-circle), polyadenylation signal (black triangle), primer binding site (black circle), and polypurine tract (black diamond) in the IGR (black line) are shown for eRTBVL/eRTBVL2 and ePVCVL/ePVCVL2. Consensus sequences of eRTBVL-X, eRTBVL2, ePVCVL, and ePVCVL2 constructed from the *O*. *sativa* genome were used for predictions.
(PDF)

S6 Fig Phylogenetic relationships of other homologous regions between eRTBVL and eRTBVL2. Maximum-likelihood (ML) trees were constructed based on ORF1, MP, CP, and PR domains (with alignment lengths of 438, 1407, 992, and 1260 nt, respectively). Bootstrap support values greater than 60% based on 1,000 replicates are shown above branches of each midpoint-rooted tree. Scale bars represent evolutionary distances in terms of substitutions per site. Sequences corresponding to different eRTBVL groups [30] are indicated by different background colors (topologies of the four phylogenetic trees show local variations due to the recombination between the viral lineages of eRTBVL groups previously reported [30]). Red circles indicate sequences corresponding to eRTBVL2.
(PDF)

S7 Fig Results of recombination analyses of the viral sequences of endogenous PRVs. Recombination analyses were performed for eRTBVL and eRTBVL2 (**A**), *Oryza* groups of ePVCVL and ePVCVL2 in the genomes of *O*. *sativa* (**B**) and other *Oryza* species (**C**), and non-*Oryza* groups of ePVCVL and ePVCVL2 in the *S*. *bicolor* genome (**D**), respectively. Different alignments for the analyses were produced because of the different lengths of examined segments having truncations. Recombination events detected with significant probabilities by more than three methods are summarized in the tables on the left. NS, not significant. Because of high sequence similarity, suggested breakpoint boundaries and possible parental sequences are variable and ambiguous for some recombination events. Recombination events were checked using BootScan plots shown on the right. In these plots, the vertical axis indicates the supporting percentage of pairwise distance measurements based on 100 bootstrap replicates, and the horizontal axis indicates relative positions in the alignments. Bars at the top of each plot indicate informative sites in each alignment. Red arrows mark the beginning site of IGRs.
(PDF)

S8 Fig Phylogenetic relationships of eRTBVL2 and ePVCVL segments based on the SFKTE domain. The ML tree, generated using amino acid sequences of the SFKTE domain (alignment length = 522 amino acids) of eRTBVL2 and ePVCVL segments in the *O*. *sativa* genome (excluding highly truncated sequences), was rooted using the oldest known ePVCVL segment (Fig 2C, S4 and S5 Tables). Bootstrap support values greater than 60% based on 1,000 replicates are shown as percentages above branches. The scale bar represents evolutionary distance in terms of substitutions per site. eRTBVL2, young ePVCVL, older ePVCVL, oldest ePVCVL, and undetermined ePVCVL segments are shown in red, purple, green, blue, and gray, respectively (details in S4 and S5 Tables). The eRTBVL2 segments related to eRTBVL-X group are shown with an orange background, and those related to other eRTBVL groups are indicated by a yellow background. YES, segments are present in the genome of *japonica*/*indica* subspecies of *O*. *sativa*; NO, segments are absent from the corresponding genomic locus or their status could not be determined because of missing genomic data.
(PDF)

S9 Fig Genomic PCR and Sanger sequencing validation of the presence/absence of representative ePVCVL and ePVCVL2 segments (orthologous loci) in *Oryza* genomes. The PCR strategy used to detect the presence/absence of a given ePVCVL or ePVCVL2 locus is shown at the top. Blue arrows indicate primers used for PCR detection. PCR and sequencing results are summarized in the table (+, presence; −, absence). Accessions of *Oryza* species used in the analysis are indicated in parentheses. Primer information is given in S6 Table.
(PDF)

S10 Fig Phylogenetic relationship of IGR sequences of ePVCVL and ePVCVL2 in *Oryza* genomes. The ML tree of IGR sequences (alignment length = 722 nt) is midpoint-rooted for display purposes. Bootstrap support values greater than 60% based on 1,000 replicates are shown as percentages above branches. Highly truncated sequences were excluded from the analysis. Scale bars represent evolutionary distances in terms of nucleotide substitutions per site. ePVCVL and ePVCVL2 sequences are indicated by green and blue, respectively (details in S3 Table). The respective orthologous sequence sets of the oldest known ePVCVL, oldest ePVCVL2, and older ePVCVL2 segments in *Oryza* genomes (details in S4 and S5 Tables) are indicated by purple, red, and orange backgrounds, respectively. Tanglegram of *Oryza* species and endogenous PRVs indicates the corresponding viral hosts.
(PDF)

S11 Fig Sequence comparisons of IGRs between *Oryza* and non-*Oryza* ePVCVL/ePVCVL2 groups. Pairwise alignments were generated between ePVCVL *Oryza* and non-*Oryza* groups (**A**) and between ePVCVL2 *Oryza* and non-*Oryza* groups (**B**) using consensus sequences constructed from *O*. *sativa* and *S*. *bicolor* genomes, respectively. Identical sites are indicated by black backgrounds. The relative position of the first nucleotide in each row of the alignment is given in parentheses. Red arrows, straight lines, and wavy lines indicate predicted strand discontinuity (i.e., the start of the minus-strand 5′-terminal), primer binding sites, and polypurine tracts, respectively.
(PDF)

S1 Table Information on grass genomes investigated in this study. (PDF)

S2 Table Identification of functional domains in protein sequences of ePVCVL and ePVCVL2. (PDF)

S3 Table Detailed information on ePVCVL and ePVCVL2 segments in grass genomes. (PDF)

S4 Table Mapping of ePVCVL and ePVCVL2 loci in the *O*. *sativa* genome to other *Oryza* genomes. (PDF)

S5 Table Mapping of ePVCVL and ePVCVL2 loci in the genomes of *O*. *glaberrima*, *O*. *glumaepatula*, *O*. *longistaminata*, *O*. *meridionalis* and *O*. *punctata* to the genome of *O*. *sativa*. (PDF)

S6 Table Information on the primers used in this study. (PDF)

S1 Dataset Amino acid alignments of all known types of RT-like domains. (TXT)

S2 Dataset Coordinates of eRTBVL2, ePVCVL, and ePVCVL2 sequences and their genes/regions in grass genomes (BED format). (ZIP)

S3 Dataset Sequence alignments used for phylogenetic analyses. (TXT)

S4 Dataset Sequence alignments used for recombination analyses. (TXT)
