# Supplementary material for: Genomic fossils reveal adaptation of non-autonomous pararetroviruses driven by concerted evolution of noncoding regulatory sequences
Source: PLoS Pathog. 2017 Jun 29;13(6):e1006413. doi: 10.1371/journal.ppat.1006413 (PMC5491270; doi:10.1371/journal.ppat.1006413)
Supplement: S1 Fig — Blue, light green, and dark green indicate eRTBVL2, eRTBVL-X, and ambiguous (eRTBVL2 or eRTBVL-X) sequences, respectively. The structures of eRTBVL2 and eRTBVL-X sequences are marked with red fonts and their lengths are drawn roughly to scale. These eRTBVL2 segments were formerly undistinguished from eRTBVL-X segments in the eRTBVL-X cluster [30]. (PDF) [file ppat.1006413.s001.pdf]

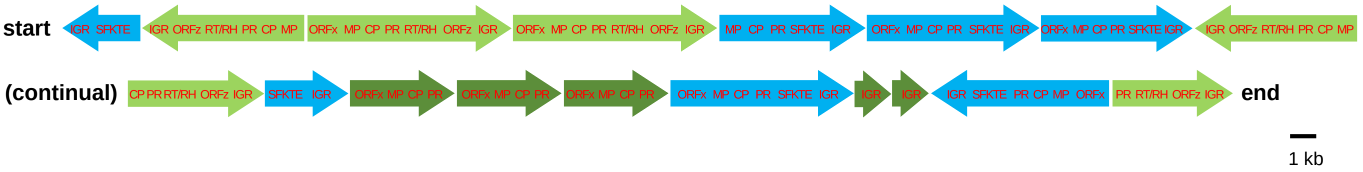

S1 Fig. Clustering of eRTBVL2 and eRTBVL-X sequences on chromosome 8 of the *Oryza sativa* genome. Blue, light green, and dark green indicate eRTBVL2, eRTBVL-X, and ambiguous (eRTBVL2 or eRTBVL-X) sequences, respectively. The structures of eRTBVL2 and eRTBVL-X sequences are marked with red fonts and their lengths are drawn roughly to scale. These eRTBVL2 segments were formerly undistinguished from eRTBVL-X segments in the eRTBVL-X cluster [30].
