# Supplementary material for: Genomic fossils reveal adaptation of non-autonomous pararetroviruses driven by concerted evolution of noncoding regulatory sequences
Source: PLoS Pathog. 2017 Jun 29;13(6):e1006413. doi: 10.1371/journal.ppat.1006413 (PMC5491270; doi:10.1371/journal.ppat.1006413)
Supplement: S2 Fig — The results of recombination analyses of the viral genomes of eRTBVL, eRTBVL2, and ePVCVL are shown at the top. The alignment for the analyses was made using viral consensus sequences constructed from the O. sativa genome (eRTBVL consensus sequence GenBank accession number: BR001199.1). The table in the upper left hand corner summarizes the recombination events detected by different methods. NS, not significant. Recombination events were checked using the BootScan plot shown on the right. In the plot, the vertical axis indicates the supporting percentage of pair-wise distance measurements based on 100 bootstrap replicates, and the horizontal axis indicates the relative position in the alignment. The bar at the top of the plot indicates informative sites in the alignment. The alignment of eRTBVL, eRTBVL2, and ePVCVL sequences at possible recombination breakpoints of their viral genomes is shown at the bottom. Segment names are shown to the left of the alignment, and segments with large deletions are highlighted in gray. Selected regions of eRTBVL2 raw (non-consensus) sequences are displayed and aligned to the corresponding regions of eRTBVL and ePVCVL consensus sequences. Regions that are highly similar between eRTBVL and eRTBVL2 sequences are indicated by red lines, while those between ePVCVL and eRTBVL2 sequences are framed in black. Suggested recombination breakpoints are marked by arrows. The microhomologous region is indicated by a yellow line. Forward slashes and hyphens represent sequence omissions and aligned gaps, respectively. (PDF) [file ppat.1006413.s002.pdf]

eRTBVL2

Methods	Breakpoint		Parent		Probability
	Left	Right	Major	Minor	
RDP	4724	7108	eRTBVL	ePVCVL	**7.18E-309**
GENECONV	4724	7108	eRTBVL	ePVCVL	4.03E-308
BootScan	4724	7108	eRTBVL	ePVCVL	2.32E-301
MaxChi	4724	7108	eRTBVL	ePVCVL	2.40E-67
Chimaera	4724	7108	eRTBVL	ePVCVL	6.38E-67
SiScan	NS	NS	NS	NS	NS

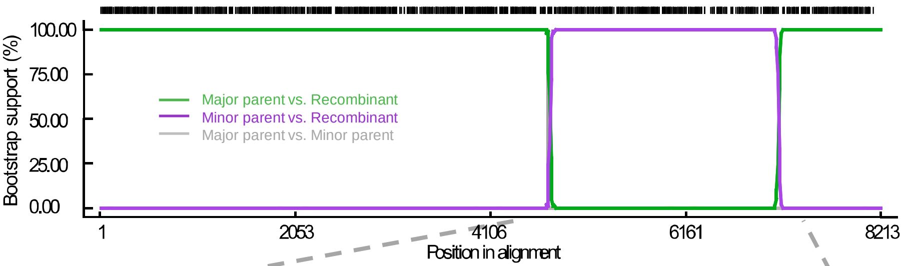

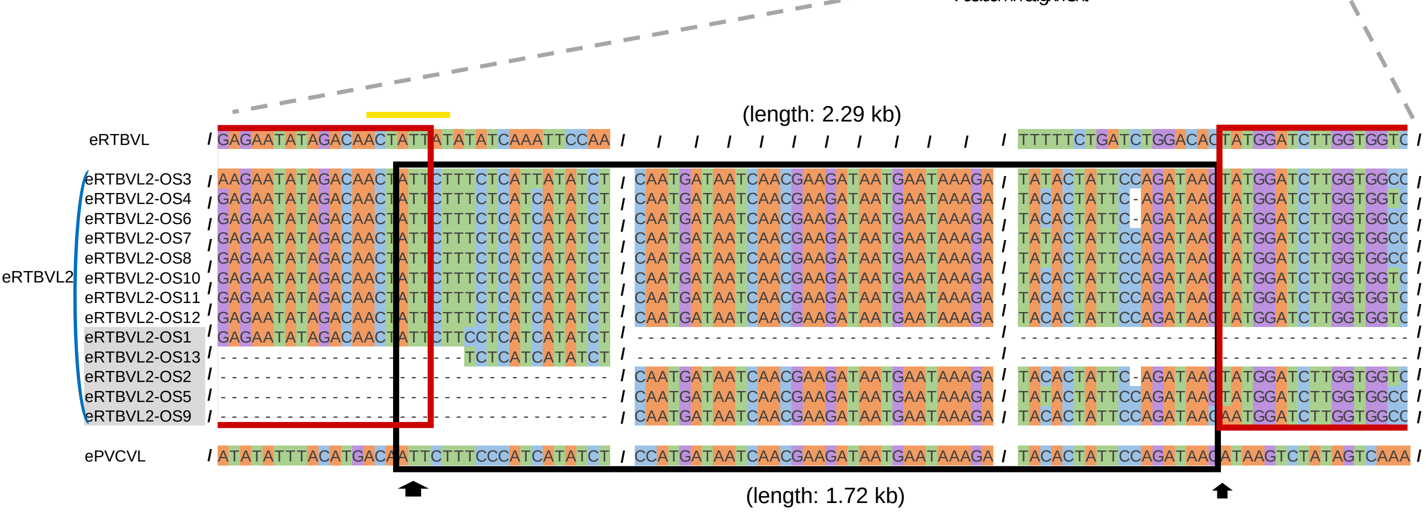

S2 Fig. Recombinational origin of the virus of eRTBVL2. The results of recombination analyses of the viral genomes of eRTBVL, eRTBVL2, and ePVCVL are shown at the top. The alignment for the analyses was made using viral consensus sequences constructed from the *O. sativa* genome (eRTBVL consensus sequence GenBank accession number: BR001199.1). The table in the upper left hand corner summarizes the recombination events detected by different methods. NS, not significant. Recombination events were checked using the BootScan plot shown on the right. In the plot, the vertical axis indicates the supporting percentage of pair-wise distance measurements based on 100 bootstrap replicates, and the horizontal axis indicates the relative position in the alignment. The bar at the top of the plot indicates informative sites in the alignment. The alignment of eRTBVL, eRTBVL2, and ePVCVL sequences at possible recombination breakpoints of their viral genomes is shown at the bottom. Segment names are shown to the left of the alignment, and segments with large deletions are highlighted in gray. Selected regions of eRTBVL2 raw (non-consensus) sequences are displayed and aligned to the corresponding regions of eRTBVL and ePVCVL consensus sequences. Regions that are highly similar between eRTBVL and eRTBVL2 sequences are indicated by red lines, while those between ePVCVL and eRTBVL2 sequences are framed in black. Suggested recombination breakpoints are marked by arrows. The microhomologous region is indicated by a yellow line. Forward slashes and hyphens represent sequence omissions and aligned gaps, respectively.
