# Supplementary material for: Genomic fossils reveal adaptation of non-autonomous pararetroviruses driven by concerted evolution of noncoding regulatory sequences
Source: PLoS Pathog. 2017 Jun 29;13(6):e1006413. doi: 10.1371/journal.ppat.1006413 (PMC5491270; doi:10.1371/journal.ppat.1006413)
Supplement: S3 Fig — Viral genomes were reconstructed from the genomes of O. sativa, O. glaberrima, O. punctata, and Sorghum bicolor, for which multiple long ePVCVL and ePVCVL2 segments were available for reconstruction. Open reading frames (ORFs) are represented by arrows, and predicted domains are outlined in different colors (white for functionally unknown regions). Intergenic regions (IGRs) are represented by black curved lines. Black stars represent the zinc finger motif in the CP domain, and black dots and diamonds indicate primer binding sites and polypurine tracts, respectively. Segment sequences used in reconstructions are represented by blue curved lines in the outer portions of the viral genomic structures and are matched to the corresponding positions of viral genomes (detailed segment information is available in S3 Table). (PDF) [file ppat.1006413.s003.pdf]

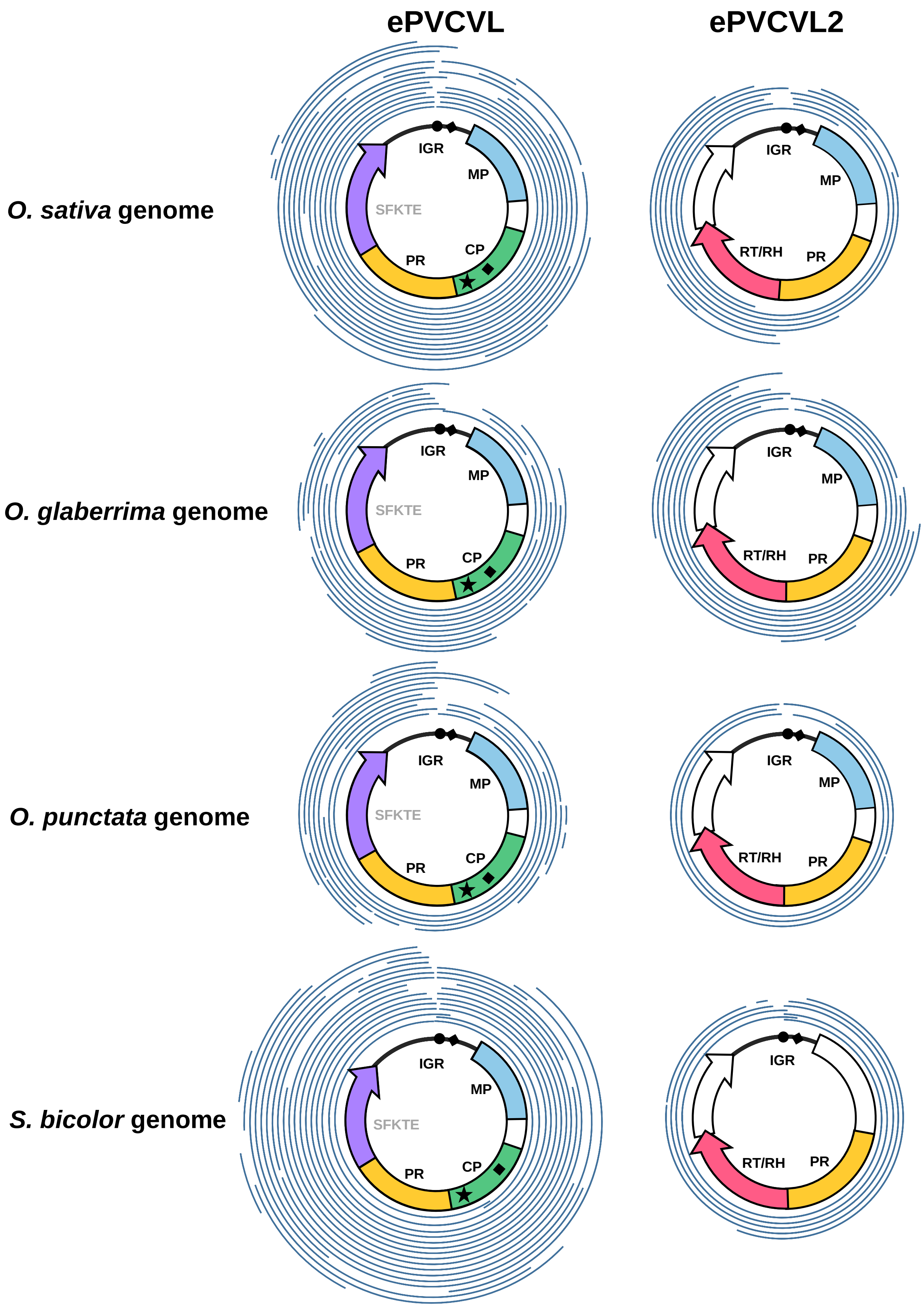

S3 Fig. Reconstruction of the ancestral virus circular genomes of ePVCVL and ePVCVL2 sequences from different grass genomes. Viral genomes were reconstructed from the genomes of *O. sativa*, *O. glaberrima*, *O. punctata*, and *Sorghum bicolor*, for which multiple long ePVCVL and ePVCVL2 segments were available for reconstruction. Open reading frames (ORFs) are represented by arrows, and predicted domains are outlined in different colors (white for functionally unknown regions). Intergenic regions (IGRs) are represented by black curved lines. Black stars represent the zinc finger motif in the CP domain, and black dots and diamonds indicate primer binding sites and polypurine tracts, respectively. Segment sequences used in reconstructions are represented by blue curved lines in the outer portions of the viral genomic structures and are matched to the corresponding positions of viral genomes (detailed segment information is available in S3 Table).
