# Supplementary material for: Genomic fossils reveal adaptation of non-autonomous pararetroviruses driven by concerted evolution of noncoding regulatory sequences
Source: PLoS Pathog. 2017 Jun 29;13(6):e1006413. doi: 10.1371/journal.ppat.1006413 (PMC5491270; doi:10.1371/journal.ppat.1006413)
Supplement: S4 Fig — Portions of sequence alignments are shown for conserved motifs in MP (A), CP (B), PR (C), and RT/RH (D) domains. Each conserved motif is indicated by a red line. Slashes represent sequence omissions. (PDF) [file ppat.1006413.s004.pdf]

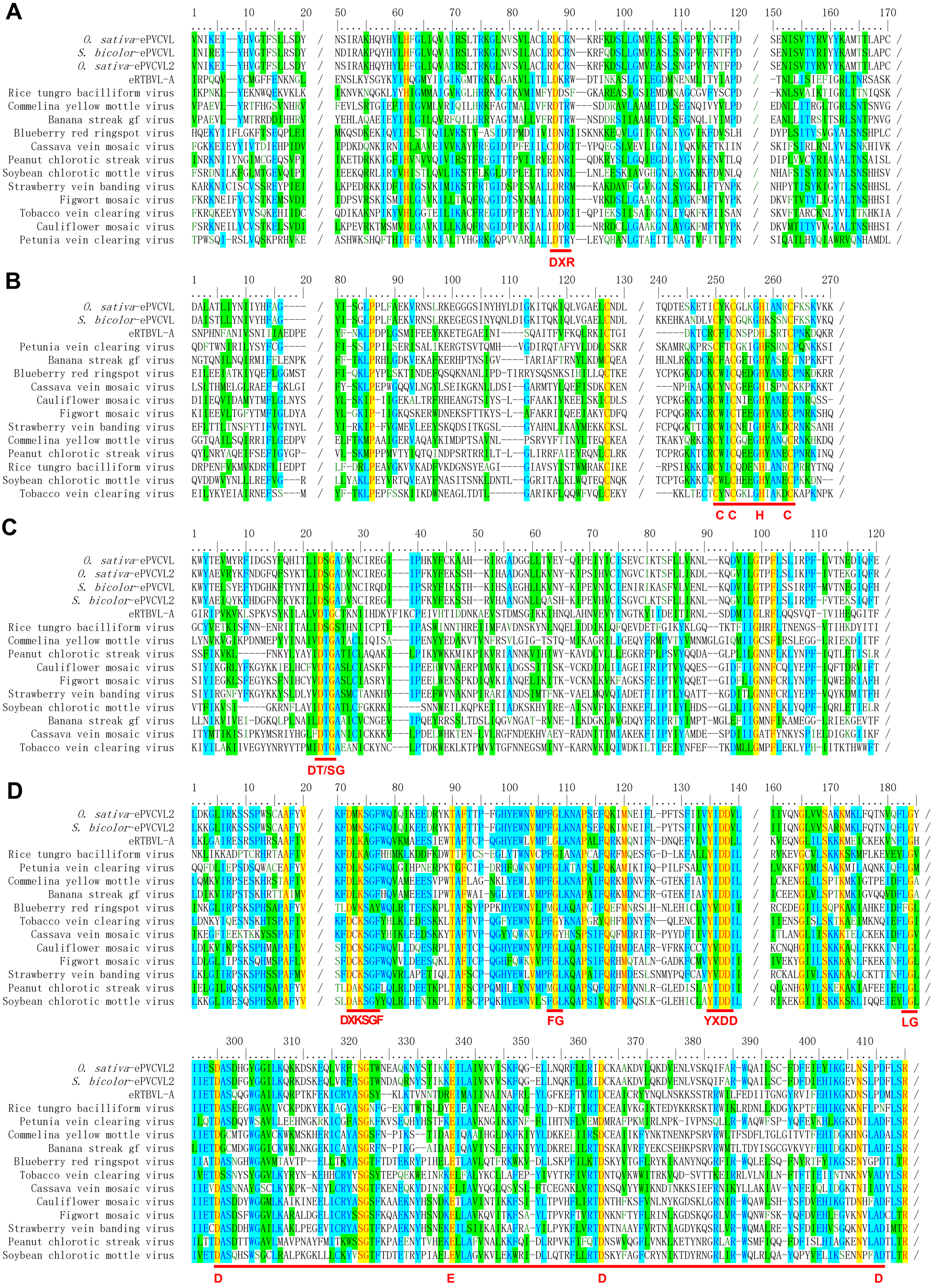

S4 Fig. Motif alignments for the four essential domains of grass endogenous PRVs and various extant PRVs. Portions of sequence alignments are shown for conserved motifs in MP (**A**), CP (**B**), PR (**C**), and RT/RH (**D**) domains. Each conserved motif is indicated by a red line. Slashes represent sequence omissions.
