# Supplementary material for: Genomic fossils reveal adaptation of non-autonomous pararetroviruses driven by concerted evolution of noncoding regulatory sequences
Source: PLoS Pathog. 2017 Jun 29;13(6):e1006413. doi: 10.1371/journal.ppat.1006413 (PMC5491270; doi:10.1371/journal.ppat.1006413)
Supplement: S5 Fig — The putative short ORF (blue box), stem-loop structure (green line), promoter (orange semi-circle), polyadenylation signal (black triangle), primer binding site (black circle), and polypurine tract (black diamond) in the IGR (black line) are shown for eRTBVL/eRTBVL2 and ePVCVL/ePVCVL2. Consensus sequences of eRTBVL-X, eRTBVL2, ePVCVL, and ePVCVL2 constructed from the O. sativa genome were used for predictions. (PDF) [file ppat.1006413.s005.pdf]

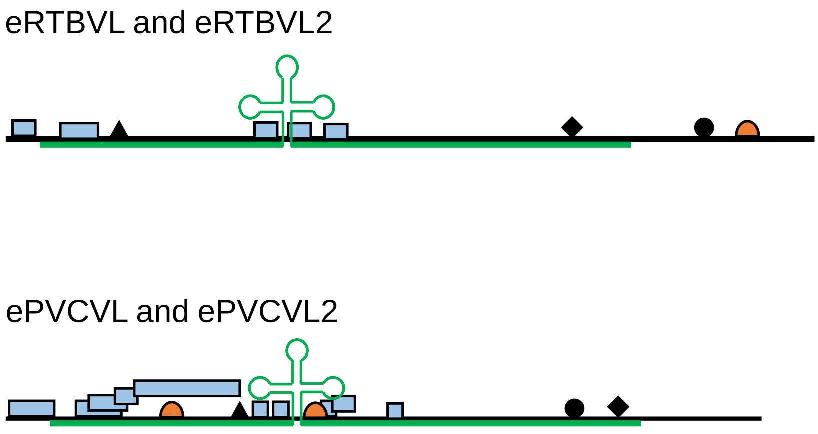

S5 Fig. Predicted elements of noncoding regulatory sequences in IGRs. The putative short ORF (blue box), stem-loop structure (green line), promoter (orange semi-circle), polyadenylation signal (black triangle), primer binding site (black circle), and polypurine tract (black diamond) in the IGR (black line) are shown for eRTBVL/eRTBVL2 and ePVCVL/ePVCVL2. Consensus sequences of eRTBVL-X, eRTBVL2, ePVCVL, and ePVCVL2 constructed from the *O. sativa* genome were used for predictions.
