# Supplementary material for: Genomic fossils reveal adaptation of non-autonomous pararetroviruses driven by concerted evolution of noncoding regulatory sequences
Source: PLoS Pathog. 2017 Jun 29;13(6):e1006413. doi: 10.1371/journal.ppat.1006413 (PMC5491270; doi:10.1371/journal.ppat.1006413)
Supplement: S6 Fig — Maximum-likelihood (ML) trees were constructed based on ORF1, MP, CP, and PR domains (with alignment lengths of 438, 1407, 992, and 1260 nt, respectively). Bootstrap support values greater than 60% based on 1,000 replicates are shown above branches of each midpoint-rooted tree. Scale bars represent evolutionary distances in terms of substitutions per site. Sequences corresponding to different eRTBVL groups [30] are indicated by different background colors (topologies of the four phylogenetic trees show local variations due to the recombination between the viral lineages of eRTBVL groups previously reported [30]). Red circles indicate sequences corresponding to eRTBVL2. (PDF) [file ppat.1006413.s006.pdf]

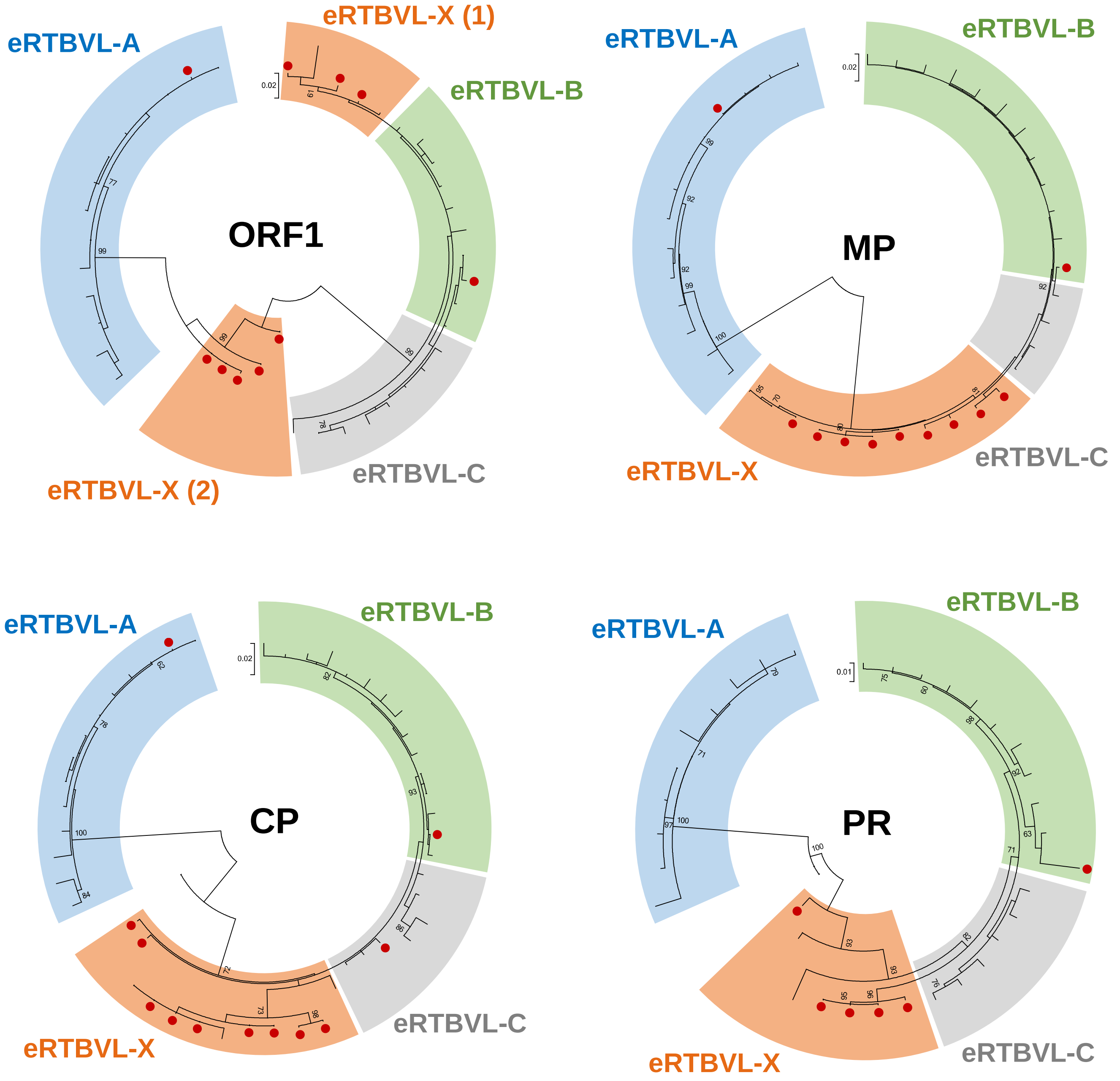

S6 Fig. Phylogenetic relationships of other homologous regions between eRTBVL and eRTBVL2. Maximum-likelihood (ML) trees were constructed based on ORF1, MP, CP, and PR domains (with alignment lengths of 438, 1407, 992, and 1260 nt, respectively). Bootstrap support values greater than 60% based on 1,000 replicates are shown above branches of each midpoint-rooted tree. Scale bars represent evolutionary distances in terms of substitutions per site. Sequences corresponding to different eRTBVL groups [30] are indicated by different background colors (topologies of the four phylogenetic trees show local variations due to the recombination between the viral lineages of eRTBVL groups previously reported [30]). Red circles indicate sequences corresponding to eRTBVL2.
