# Supplementary material for: Genomic fossils reveal adaptation of non-autonomous pararetroviruses driven by concerted evolution of noncoding regulatory sequences
Source: PLoS Pathog. 2017 Jun 29;13(6):e1006413. doi: 10.1371/journal.ppat.1006413 (PMC5491270; doi:10.1371/journal.ppat.1006413)
Supplement: S7 Fig — Recombination analyses were performed for eRTBVL and eRTBVL2 (A), Oryza groups of ePVCVL and ePVCVL2 in the genomes of O. sativa (B) and other Oryza species (C), and non-Oryza groups of ePVCVL and ePVCVL2 in the S. bicolor genome (D), respectively. Different alignments for the analyses were produced because of the different lengths of examined segments having truncations. Recombination events detected with significant probabilities by more than three methods are summarized in the tables on the left. NS, not significant. Because of high sequence similarity, suggested breakpoint boundaries and possible parental sequences are variable and ambiguous for some recombination events. Recombination events were checked using BootScan plots shown on the right. In these plots, the vertical axis indicates the supporting percentage of pairwise distance measurements based on 100 bootstrap replicates, and the horizontal axis indicates relative positions in the alignments. Bars at the top of each plot indicate informative sites in each alignment. Red arrows mark the beginning site of IGRs. (PDF) [file ppat.1006413.s007.pdf]

A

eRTBVL2-OS10

Methods	Breakpoint		Parent		Probability
	Left	Right	Major	Minor	
RDP	6689	4044	eRTBVL2-OS8	eRTBVL-JaE9-1 (A2)	3.77E-178
GENECONV	6689	4044	eRTBVL2-OS8	eRTBVL-JaE9-1 (A2)	4.02E-169
BootScan	6689	4044	eRTBVL2-OS8	eRTBVL-JaE9-1 (A2)	**1.44E-181**
MaxChi	6689	4044	eRTBVL2-OS8	eRTBVL-JaE9-1 (A2)	2.25E-60
Chimaera	6689	4044	eRTBVL2-OS8	eRTBVL-JaE9-1 (A2)	6.38E-43
SiScan	6689	4044	eRTBVL2-OS8	eRTBVL-JaE9-1 (A2)	5.67E-99

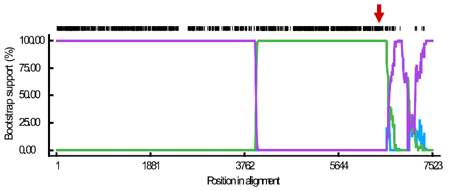

eRTBVL2-OS11

Methods	Breakpoint		Parent		Probability
	Left	Right	Major	Minor	
RDP	6580	2081	eRTBVL2-OS8	eRTBVL-JaE7-7 (A1)	1.23E-164
GENECONV	6580	2081	eRTBVL2-OS8	eRTBVL-JaE7-7 (A1)	**1.74E-166**
BootScan	6580	2081	eRTBVL2-OS8	eRTBVL-JaE7-7 (A1)	3.70E-159
MaxChi	6590	2081	eRTBVL2-OS8	eRTBVL-JaE6-5 (A1)	2.43E-42
Chimaera	6580	2081	eRTBVL2-OS8	eRTBVL-JaE7-7 (A1)	1.36E-42
SiScan	6580	2081	eRTBVL2-OS8	eRTBVL-JaE7-7 (A1)	7.17E-71

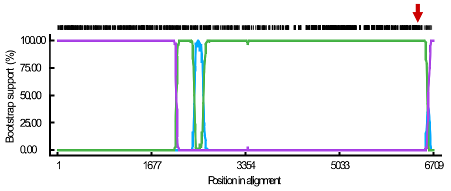

eRTBVL2-OS12

Methods	Breakpoint		Parent		Probability
	Left	Right	Major	Minor	
RDP	3366	992	eRTBVL2-OS2	eRTBVL-JaE12-9 (B)	3.87E-26
GENECONV	3366	992	eRTBVL2-OS2	eRTBVL-JaE12-9 (B)	1.82E-24
BootScan	3366	992	eRTBVL2-OS2	eRTBVL-JaE12-9 (B)	**2.67E-31**
MaxChi	NS	NS	NS	NS	NS
Chimaera	3540	938	eRTBVL2-OS10	eRTBVL-JaE12-9 (B)	3.58E-21
SiScan	NS	NS	NS	NS	NS

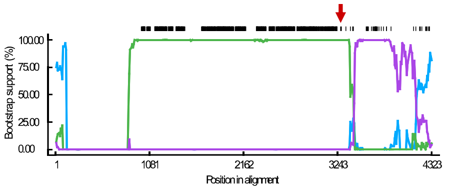

eRTBVL2-OS2

Methods	Breakpoint		Parent		Probability
	Left	Right	Major	Minor	
RDP	2442	3282	eRTBVL2-OS9	eRTBVL-JaE8-11 (X)	1.74E-08
GENECONV	NS	NS	NS	NS	NS
BootScan	2442	2785	eRTBVL2-OS9	eRTBVL-JaE8-8 (X)	**1.37E-09**
MaxChi	2442	3282	eRTBVL2-OS9	eRTBVL-JaE8-11 (X)	1.03E-05
Chimaera	NS	NS	NS	NS	NS
SiScan	NS	NS	NS	NS	NS

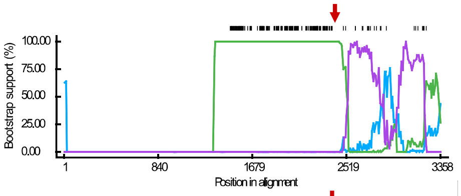

eRTBVL2-OS3

Methods	Breakpoint		Parent		Probability
	Left	Right	Major	Minor	
RDP	NS	NS	NS	NS	NS
GENECONV	2346	3282	eRTBVL2-OS9	eRTBVL-JaE8-11 (X)	6.70E-17
BootScan	2346	3282	eRTBVL2-OS9	eRTBVL-JaE8-11 (X)	**6.08E-18**
MaxChi	2346	3282	eRTBVL2-OS9	eRTBVL-JaE8-11 (X)	3.02E-08
Chimaera	2346	3282	eRTBVL2-OS9	eRTBVL-JaE8-11 (X)	4.80E-07
SiScan	NS	NS	NS	NS	NS

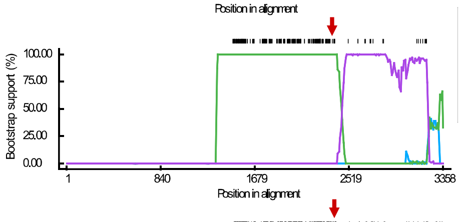

eRTBVL2-OS4

Methods	Breakpoint		Parent		Probability
	Left	Right	Major	Minor	
RDP	2452	3306	eRTBVL2-OS9	eRTBVL-JaE8-8 (X)	1.77E-11
GENECONV	2442	2949	eRTBVL2-OS2	eRTBVL-JaE8-11 (X)	1.55E-06
BootScan	2452	3247	eRTBVL2-OS9	eRTBVL-JaE8-8 (X)	**1.58E-11**
MaxChi	2442	3282	eRTBVL2-OS9	eRTBVL-JaE8-11 (X)	3.29E-05
Chimaera	2452	3247	eRTBVL2-OS9	eRTBVL-JaE8-8 (X)	2.03E-03
SiScan	NS	NS	NS	NS	NS

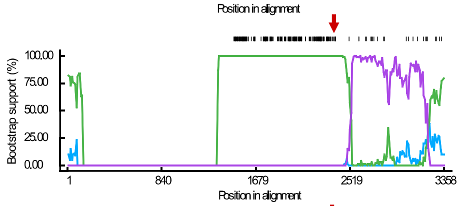

eRTBVL2-OS6

Methods	Breakpoint		Parent		Probability
	Left	Right	Major	Minor	
RDP	2356	3207	eRTBVL2-OS2	eRTBVL-JaE8-11 (X)	**2.35E-11**
GENECONV	2360	2716	eRTBVL2-OS9	eRTBVL-JaE8-11 (X)	2.71E-08
BootScan	2360	3129	eRTBVL2-OS9	eRTBVL-JaE8-11 (X)	3.33E-10
MaxChi	NS	NS	NS	NS	NS
Chimaera	NS	NS	NS	NS	NS
SiScan	NS	NS	NS	NS	NS

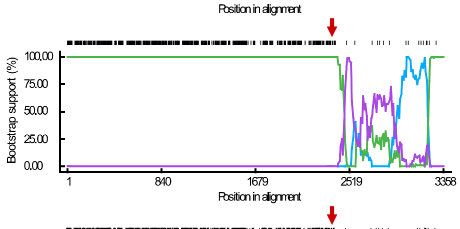

eRTBVL2-OS7

Methods	Breakpoint		Parent		Probability
	Left	Right	Major	Minor	
RDP	2356	3004	eRTBVL2-OS2	eRTBVL-JaE8-11 (X)	**4.79E-15**
GENECONV	2360	3129	eRTBVL2-OS9	eRTBVL-JaE8-11 (X)	4.06E-12
BootScan	2360	3129	eRTBVL2-OS9	eRTBVL-JaE8-11 (X)	1.27E-13
MaxChi	2283	3129	eRTBVL2-OS9	eRTBVL-JaE8-11 (X)	2.24E-03
Chimaera	NS	NS	NS	NS	NS
SiScan	NS	NS	NS	NS	NS

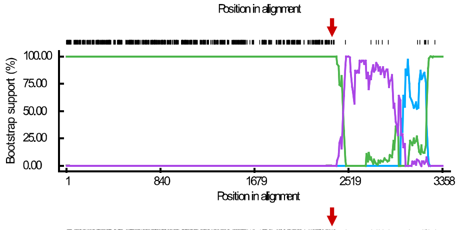

eRTBVL2-OS8

Methods	Breakpoint		Parent		Probability
	Left	Right	Major	Minor	
RDP	2356	2966	eRTBVL2-OS2	eRTBVL-JaE8-11 (X)	**4.56E-15**
GENECONV	2360	3167	eRTBVL2-OS9	eRTBVL-JaE8-11 (X)	1.28E-09
BootScan	2360	3167	eRTBVL2-OS9	eRTBVL-JaE8-11 (X)	1.59E-14
MaxChi	NS	NS	NS	NS	NS
Chimaera	NS	NS	NS	NS	NS
SiScan	2360	3167	eRTBVL2-OS9	eRTBVL-JaE8-11 (X)	1.59E-14

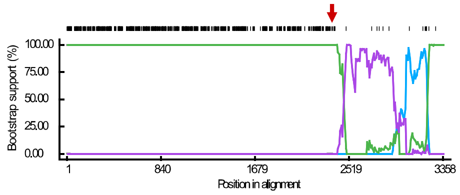

B

ePVCVL-OS2

Methods	Breakpoint		Parent		Probability
	Left	Right	Major	Minor	
RDP	738	1276	ePVCVL-OS18	ePVCVL2-OS11	3.63E-13
GENECONV	745	1276	ePVCVL-OS18	ePVCVL2-OS11	9.35E-10
BootScan	726	1276	Unknown	ePVCVL2-OS11	**9.23E-16**
MaxChi	NS	NS	NS	NS	NS
Chimaera	738	1410	ePVCVL-OS18	ePVCVL2-OS8	4.90E-03
SiScan	738	1276	ePVCVL-OS18	ePVCVL2-OS11	9.42E-09

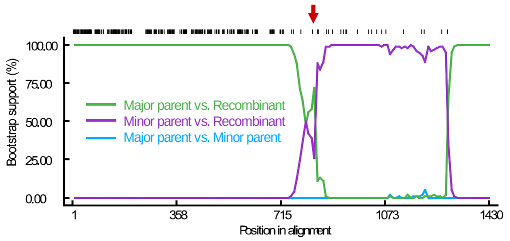

ePVCVL-OS8

Methods	Breakpoint		Parent		Probability
	Left	Right	Major	Minor	
RDP	738	1314	ePVCVL-OS18	ePVCVL2-OS11	1.56E-13
GENECONV	738	1314	ePVCVL-OS18	ePVCVL2-OS11	4.65E-11
BootScan	726	1348	Unknown	ePVCVL2-OS11	**7.26E-16**
MaxChi	NS	NS	NS	NS	NS
Chimaera	738	1368	ePVCVL-OS18	ePVCVL2-OS8	1.35E-03
SiScan	738	1314	ePVCVL-OS18	ePVCVL2-OS11	2.70E-09

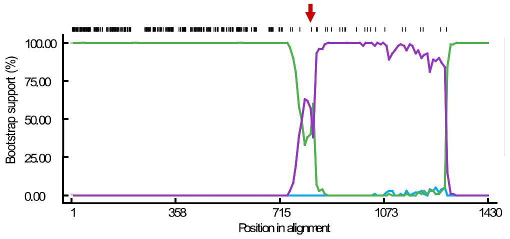

ePVCVL-OS21

Methods	Breakpoint		Parent		Probability
	Left	Right	Major	Minor	
RDP	738	1070	ePVCVL-OS18	ePVCVL2-OS11	**1.28E-08**
GENECONV	745	943	ePVCVL-OS18	ePVCVL2-OS11	9.58E-08
BootScan	726	1010	Unknown	ePVCVL2-OS11	2.83E-06
MaxChi	NS	NS	NS	NS	NS
Chimaera	800	1375	ePVCVL-OS5	ePVCVL2-OS8	2.18E-04
SiScan	738	1070	ePVCVL-OS18	ePVCVL2-OS11	1.62E-07

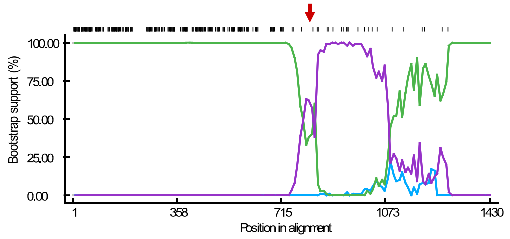

ePVCVL2-OS5

Methods	Breakpoint		Parent		Probability
	Left	Right	Major	Minor	
RDP	739	1290	Unknown	ePVCVL-OS8	2.23E-09
GENECONV	787	1000	Unknown	ePVCVL-OS8	6.16E-07
BootScan	787	1396	Unknown	ePVCVL-OS8	**7.97E-11**
MaxChi	NS	NS	NS	NS	NS
Chimaera	NS	NS	NS	NS	NS
SiScan	NS	NS	NS	NS	NS

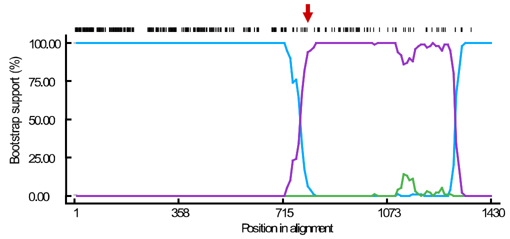

ePVCVL2-OS8

Methods	Breakpoint		Parent		Probability
	Left	Right	Major	Minor	
RDP	735	1429	Unknown	ePVCVL-OS8	3.40E-18
GENECONV	764	1429	Unknown	ePVCVL-OS8	9.18E-15
BootScan	764	1429	Unknown	ePVCVL-OS8	**1.09E-18**
MaxChi	NS	NS	NS	NS	NS
Chimaera	NS	NS	NS	NS	NS
SiScan	NS	NS	NS	NS	NS

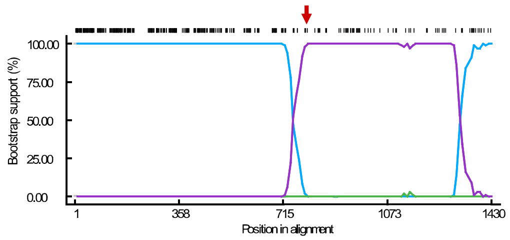

ePVCVL2-OS10

Methods	Breakpoint		Parent		Probability
	Left	Right	Major	Minor	
RDP	701	1167	Unknown	ePVCVL-OS8	7.73E-14
GENECONV	723	1388	Unknown	ePVCVL-OS8	5.17E-10
BootScan	725	1341	Unknown	ePVCVL-OS8	**1.51E-14**
MaxChi	NS	NS	NS	NS	NS
Chimaera	NS	NS	NS	NS	NS
SiScan	NS	NS	NS	NS	NS

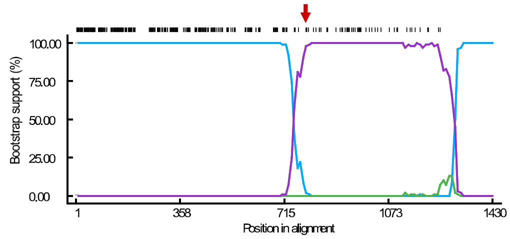

ePVCVL2-OS11

Methods	Breakpoint		Parent		Probability
	Left	Right	Major	Minor	
RDP	722	1346	Unknown	ePVCVL-OS8	1.20E-21
GENECONV	722	1346	Unknown	ePVCVL-OS8	6.45E-23
BootScan	722	1346	Unknown	ePVCVL-OS8	**2.90E-23**
MaxChi	NS	NS	NS	NS	NS
Chimaera	726	1340	ePVCVL2-OS5	ePVCVL-OS2	7.58E-06
SiScan	NS	NS	NS	NS	NS

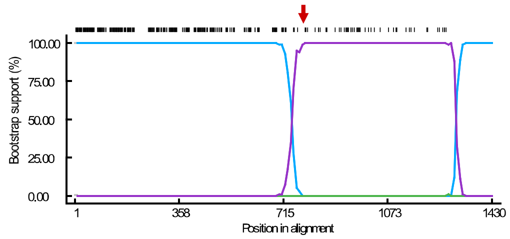

C

ePVCVL-OG6

Methods	Breakpoint		Parent		Probability
	Left	Right	Major	Minor	
RDP	665	1193	Unknown	ePVCVL2-OG12	2.03E-05
GENECONV	NS	NS	NS	NS	NS
BootScan	692	1193	Unknown	ePVCVL2-OG12	3.95E-05
MaxChi	546	1194	Unknown	ePVCVL2-OG3	**1.27E-06**
Chimaera	577	1194	Unknown	ePVCVL2-OG12	9.64E-04
SiScan	NS	NS	NS	NS	NS

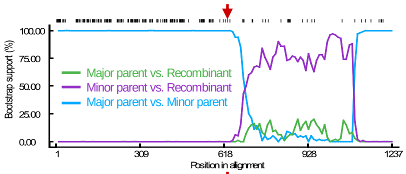

ePVCVL-OG8

Methods	Breakpoint		Parent		Probability
	Left	Right	Major	Minor	
RDP	577	1214	Unknown	ePVCVL2-OG12	**1.57E-10**
GENECONV	623	1082	ePVCVL-OG11	ePVCVL2-OG12	1.01E-06
BootScan	577	1214	Unknown	ePVCVL2-OG12	1.05E-09
MaxChi	577	1198	Unknown	ePVCVL2-OG3	1.06E-07
Chimaera	577	1214	Unknown	ePVCVL2-OG12	2.32E-06
SiScan	768	1218	ePVCVL-OG18	ePVCVL2-OG12	7.33E-03

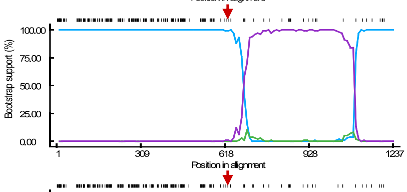

ePVCVL-OG9

Methods	Breakpoint		Parent		Probability
	Left	Right	Major	Minor	
RDP	620	1193	Unknown	ePVCVL2-OG12	**3.82E-07**
GENECONV	NS	NS	NS	NS	NS
BootScan	692	1193	Unknown	ePVCVL2-OG12	9.89E-06
MaxChi	NS	NS	NS	NS	NS
Chimaera	577	1215	Unknown	ePVCVL2-OG12	1.40E-04
SiScan	NS	NS	NS	NS	NS

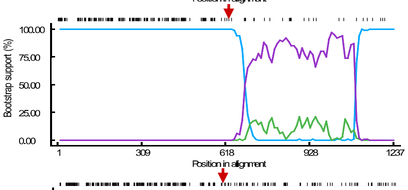

ePVCVL2-OG1

Methods	Breakpoint		Parent		Probability
	Left	Right	Major	Minor	
RDP	616	1338	Unknown	ePVCVL-OG8	2.62E-07
GENECONV	NS	NS	NS	NS	NS
BootScan	616	1338	Unknown	ePVCVL-OG8	**3.19E-18**
MaxChi	NS	NS	NS	NS	NS
Chimaera	616	1338	Unknown	ePVCVL-OG8	6.25E-14
SiScan	NS	NS	NS	NS	NS

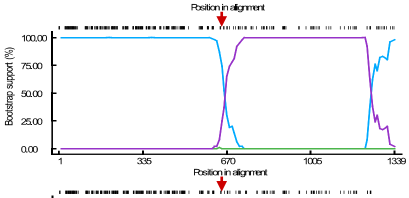

ePVCVL2-OG2

Methods	Breakpoint		Parent		Probability
	Left	Right	Major	Minor	
RDP	616	1291	Unknown	ePVCVL-OG8	2.97E-19
GENECONV	NS	NS	NS	NS	NS
BootScan	616	1291	Unknown	ePVCVL-OG8	**2.57E-19**
MaxChi	NS	NS	NS	NS	NS
Chimaera	616	1291	Unknown	ePVCVL-OG8	7.82E-13
SiScan	NS	NS	NS	NS	NS

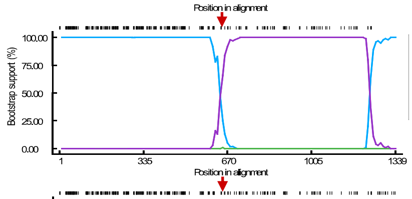

ePVCVL2-OG3

Methods	Breakpoint		Parent		Probability
	Left	Right	Major	Minor	
RDP	616	1338	Unknown	ePVCVL-OG8	**3.66E-18**
GENECONV	NS	NS	NS	NS	NS
BootScan	616	1338	Unknown	ePVCVL-OG8	6.39E-18
MaxChi	NS	NS	NS	NS	NS
Chimaera	616	1338	Unknown	ePVCVL-OG8	3.77E-13
SiScan	NS	NS	NS	NS	NS

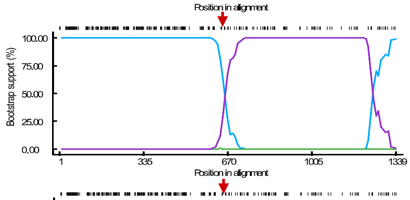

ePVCVL2-OG8

Methods	Breakpoint		Parent		Probability
	Left	Right	Major	Minor	
RDP	616	1338	Unknown	ePVCVL-OG8	1.84E-18
GENECONV	NS	NS	NS	NS	NS
BootScan	616	1338	Unknown	ePVCVL-OG8	**1.11E-18**
MaxChi	NS	NS	NS	NS	NS
Chimaera	616	1338	Unknown	ePVCVL-OG8	2.35E-13
SiScan	NS	NS	NS	NS	NS

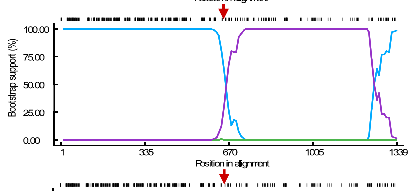

ePVCVL2-OG12

Methods	Breakpoint		Parent		Probability
	Left	Right	Major	Minor	
RDP	616	1339	Unknown	ePVCVL-OG8	**6.87E-23**
GENECONV	NS	NS	NS	NS	NS
BootScan	616	1339	Unknown	ePVCVL-OG8	4.05E-22
MaxChi	650	1284	ePVCVL2-OG3	ePVCVL-OG8	6.23E-04
Chimaera	616	1339	Unknown	ePVCVL-OG8	3.50E-14
SiScan	NS	NS	NS	NS	NS

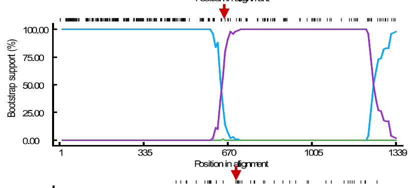

ePVCVL-OP16

Methods	Breakpoint		Parent		Probability
	Left	Right	Major	Minor	
RDP	NS	NS	NS	NS	NS
GENECONV	1118	1335	ePVCVL-OP15	Unknown	1.53E-03
BootScan	1118	1335	ePVCVL-OP15	Unknown	**4.06E-04**
MaxChi	NS	NS	NS	NS	NS
Chimaera	NS	NS	NS	NS	NS
SiScan	1118	1335	ePVCVL-OP15	Unknown	4.23E-03

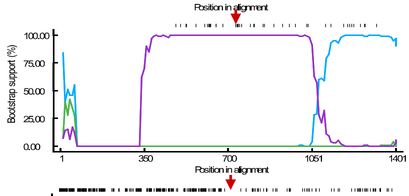

ePVCVL2-OP1

Methods	Breakpoint		Parent		Probability
	Left	Right	Major	Minor	
RDP	726	1388	ePVCVL2-OP2	ePVCVL-OP13	4.48E-10
GENECONV	NS	NS	NS	NS	NS
BootScan	726	1388	ePVCVL2-OP2	ePVCVL-OP13	2.55E-09
MaxChi	673	1388	ePVCVL2-OP2	ePVCVL-OP21	**1.25E-11**
Chimaera	726	1388	ePVCVL2-OP2	ePVCVL-OP13	3.50E-09
SiScan	NS	NS	NS	NS	NS

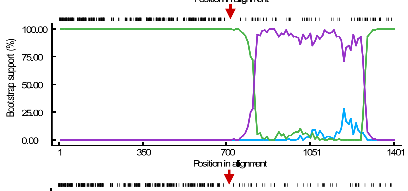

ePVCVL2-OP4

Methods	Breakpoint		Parent		Probability
	Left	Right	Major	Minor	
RDP	714	1386	ePVCVL2-OP2	ePVCVL-OP9	**1.33E-13**
GENECONV	1247	1386	ePVCVL2-OP2	ePVCVL-OP9	1.96E-08
BootScan	1021	1386	ePVCVL2-OP2	ePVCVL-OP13	2.27E-03
MaxChi	714	1386	ePVCVL2-OP2	ePVCVL-OP9	2.27E-10
Chimaera	753	1386	Unknown	ePVCVL-OP9	2.64E-09
SiScan	1072	1386	ePVCVL2-OP2	ePVCVL-OP9	4.15E-06

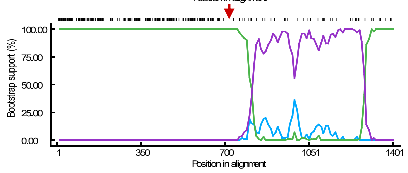

D

ePVCVL-SB1

Methods	Breakpoint		Parent		Probability
	Left	Right	Major	Minor	
RDP	675	1321	ePVCVL-SB3	ePVCVL2-SB6	1.35E-08
GENECONV	NS	NS	NS	NS	NS
BootScan	NS	NS	NS	NS	NS
MaxChi	656	1268	Unknown	ePVCVL2-SB6	2.70E-09
Chimaera	665	1414	ePVCVL-SB3	ePVCVL2-SB6	**5.39E-10**
SiScan	NS	NS	NS	NS	NS

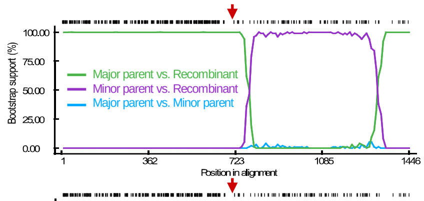

ePVCVL-SB6

Methods	Breakpoint		Parent		Probability
	Left	Right	Major	Minor	
RDP	675	1264	ePVCVL-SB3	ePVCVL2-SB6	**2.44E-10**
GENECONV	NS	NS	NS	NS	NS
BootScan	NS	NS	NS	NS	NS
MaxChi	665	1234	Unknown	ePVCVL2-SB6	3.83E-08
Chimaera	666	1398	ePVCVL-SB3	ePVCVL2-SB6	6.67E-04
SiScan	NS	NS	NS	NS	NS

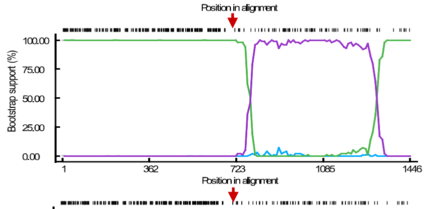

ePVCVL-SB7

Methods	Breakpoint		Parent		Probability
	Left	Right	Major	Minor	
RDP	675	1276	ePVCVL-SB3	ePVCVL2-SB6	4.93E-11
GENECONV	NS	NS	NS	NS	NS
BootScan	NS	NS	NS	NS	NS
MaxChi	656	1244	Unknown	ePVCVL2-SB6	3.38E-09
Chimaera	665	1414	ePVCVL-SB3	ePVCVL2-SB6	**4.53E-11**
SiScan	NS	NS	NS	NS	NS

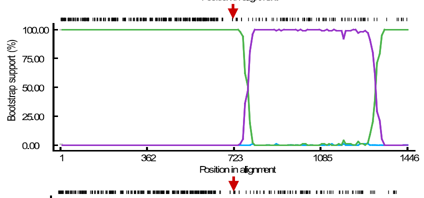

ePVCVL-SB10

Methods	Breakpoint		Parent		Probability
	Left	Right	Major	Minor	
RDP	665	1286	ePVCVL-SB3	ePVCVL2-SB6	**7.81E-12**
GENECONV	NS	NS	NS	NS	NS
BootScan	NS	NS	NS	NS	NS
MaxChi	660	1244	Unknown	ePVCVL2-SB6	1.28E-09
Chimaera	665	1428	ePVCVL-SB3	ePVCVL2-SB6	4.48E-11
SiScan	NS	NS	NS	NS	NS

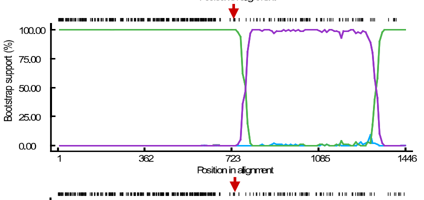

ePVCVL-SB13

Methods	Breakpoint		Parent		Probability
	Left	Right	Major	Minor	
RDP	675	1348	ePVCVL-SB3	ePVCVL2-SB6	**6.30E-10**
GENECONV	NS	NS	NS	NS	NS
BootScan	NS	NS	NS	NS	NS
MaxChi	651	1260	Unknown	ePVCVL2-SB6	6.36E-09
Chimaera	666	1402	ePVCVL-SB3	ePVCVL2-SB6	1.23E-04
SiScan	NS	NS	NS	NS	NS

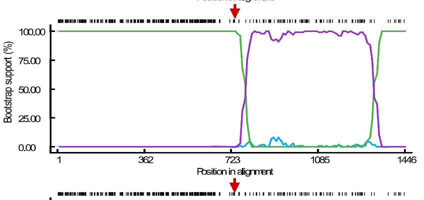

ePVCVL-SB17

Methods	Breakpoint		Parent		Probability
	Left	Right	Major	Minor	
RDP	729	1274	ePVCVL-SB3	ePVCVL2-SB6	2.20E-11
GENECONV	NS	NS	NS	NS	NS
BootScan	NS	NS	NS	NS	NS
MaxChi	656	1241	Unknown	ePVCVL2-SB6	5.21E-09
Chimaera	665	1414	ePVCVL-SB3	ePVCVL2-SB6	**1.10E-11**
SiScan	NS	NS	NS	NS	NS

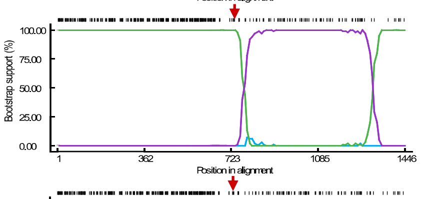

ePVCVL-SB19

Methods	Breakpoint		Parent		Probability
	Left	Right	Major	Minor	
RDP	675	1339	ePVCVL-SB3	ePVCVL2-SB6	**1.01E-10**
GENECONV	NS	NS	NS	NS	NS
BootScan	NS	NS	NS	NS	NS
MaxChi	656	1259	Unknown	ePVCVL2-SB6	2.18E-09
Chimaera	666	1398	ePVCVL-SB3	ePVCVL2-SB6	6.90E-04
SiScan	NS	NS	NS	NS	NS

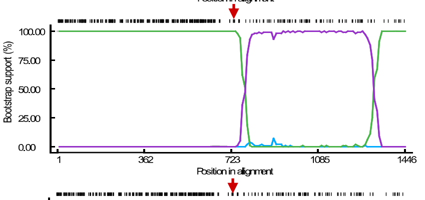

ePVCVL-SB20

Methods	Breakpoint		Parent		Probability
	Left	Right	Major	Minor	
RDP	675	1347	ePVCVL-SB3	ePVCVL2-SB6	**1.92E-10**
GENECONV	NS	NS	NS	NS	NS
BootScan	NS	NS	NS	NS	NS
MaxChi	656	1260	Unknown	ePVCVL2-SB6	5.25E-09
Chimaera	665	1414	ePVCVL-SB3	ePVCVL2-SB6	1.13E-09
SiScan	NS	NS	NS	NS	NS

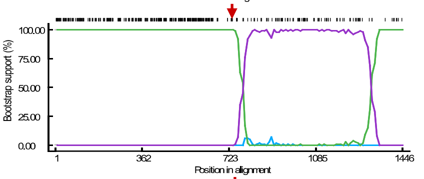

ePVCVL2-SB1

Methods	Breakpoint		Parent		Probability
	Left	Right	Major	Minor	
RDP	672	1446	Unknown	ePVCVL-SB1	**1.23E-22**
GENECONV	672	1267	Unknown	ePVCVL-SB10	2.35E-22
BootScan	672	1425	Unknown	ePVCVL-SB10	2.15E-20
MaxChi	672	1267	Unknown	ePVCVL-SB10	6.24E-14
Chimaera	672	1446	Unknown	ePVCVL-SB1	7.47E-15
SiScan	672	1438	Unknown	ePVCVL-SB6	8.09E-19

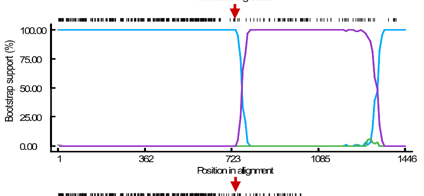

ePVCVL2-SB4

Methods	Breakpoint		Parent		Probability
	Left	Right	Major	Minor	
RDP	NS	NS	NS	NS	NS
GENECONV	850	1218	Unknown	ePVCVL-SB10	3.99E-06
BootScan	850	1218	Unknown	ePVCVL-SB17	**2.04E-10**
MaxChi	NS	NS	NS	NS	NS
Chimaera	NS	NS	NS	NS	NS
SiScan	850	1218	Unknown	ePVCVL-SB10	1.40E-05

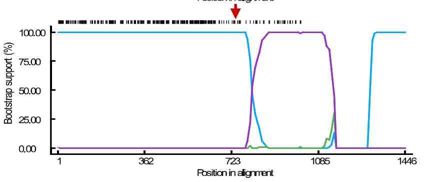

S7 Fig. Results of recombination analyses of the viral sequences of endogenous PRVs. Recombination analyses were performed for eRTBVL and eRTBVL2 (**A**), *Oryza* groups of ePVCVL and ePVCVL2 in the genomes of *O. sativa* (**B**) and other *Oryza* species (**C**), and non-*Oryza* groups of ePVCVL and ePVCVL2 in the *S. bicolor* genome (**D**), respectively**.** Different alignments for the analyses were produced because of the different lengths of examined segments having truncations. Recombination events detected with significant probabilities by more than three methods are summarized in the tables on the left. NS, not significant. Because of high sequence similarity, suggested breakpoint boundaries and possible parental sequences are variable and ambiguous for some recombination events. Recombination events were checked using BootScan plots shown on the right. In these plots, the vertical axis indicates the supporting percentage of pairwise distance measurements based on 100 bootstrap replicates, and the horizontal axis indicates relative positions in the alignments. Bars at the top of each plot indicate informative sites in each alignment. Red arrows mark the beginning site of IGRs.
