# Supplementary material for: Genomic fossils reveal adaptation of non-autonomous pararetroviruses driven by concerted evolution of noncoding regulatory sequences
Source: PLoS Pathog. 2017 Jun 29;13(6):e1006413. doi: 10.1371/journal.ppat.1006413 (PMC5491270; doi:10.1371/journal.ppat.1006413)
Supplement: S8 Fig — The ML tree, generated using amino acid sequences of the SFKTE domain (alignment length = 522 amino acids) of eRTBVL2 and ePVCVL segments in the O. sativa genome (excluding highly truncated sequences), was rooted using the oldest known ePVCVL segment (Fig 2C, S4 and S5 Tables). Bootstrap support values greater than 60% based on 1,000 replicates are shown as percentages above branches. The scale bar represents evolutionary distance in terms of substitutions per site. eRTBVL2, young ePVCVL, older ePVCVL, oldest ePVCVL, and undetermined ePVCVL segments are shown in red, purple, green, blue, and gray, respectively (details in S4 and S5 Tables). The eRTBVL2 segments related to eRTBVL-X group are shown with an orange background, and those related to other eRTBVL groups are indicated by a yellow background. YES, segments are present in the genome of japonica/indica subspecies of O. sativa; NO, segments are absent from the corresponding genomic locus or their status could not be determined because of missing genomic data. (PDF) [file ppat.1006413.s008.pdf]

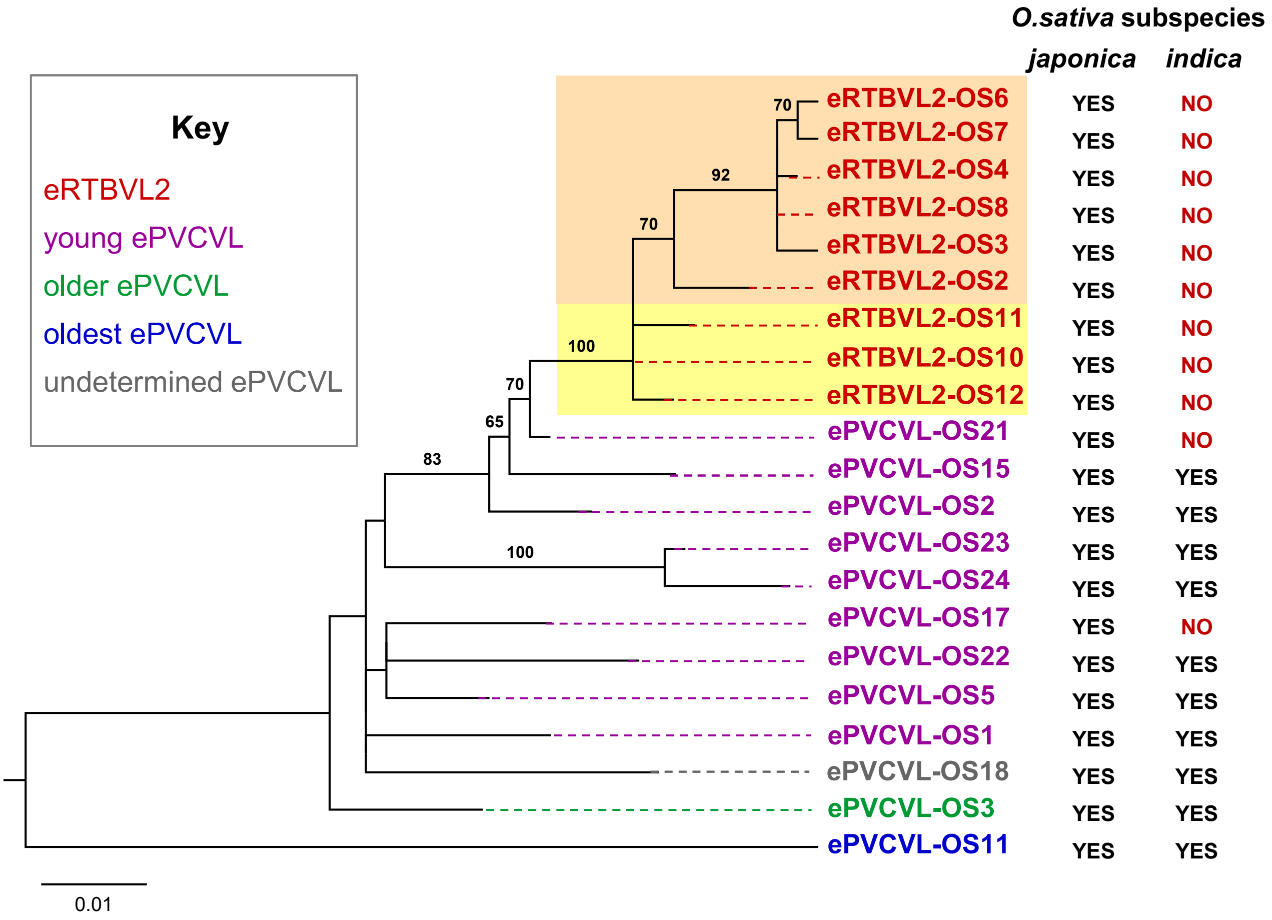

S8 Fig. Phylogenetic relationships of eRTBVL2 and ePVCVL segments based on the SFKTE domain. The ML tree, generated using amino acid sequences of the SFKTE domain (alignment length = 522 amino acids) of eRTBVL2 and ePVCVL segments in the *O. sativa* genome (excluding highly truncated sequences), was rooted using the oldest known ePVCVL segment (Fig 2C, S4 and S5 Tables). Bootstrap support values greater than 60% based on 1,000 replicates are shown as percentages above branches. The scale bar represents evolutionary distance in terms of substitutions per site. eRTBVL2, young ePVCVL, older ePVCVL, oldest ePVCVL, and undetermined ePVCVL segments are shown in red, purple, green, blue, and gray, respectively (details in S4 and S5 Tables). The eRTBVL2 segments related to eRTBVL-X group are shown with an orange background, and those related to other eRTBVL groups are indicated by a yellow background. YES, segments are present in the genome of *japonica*/*indica* subspecies of *O. sativa*; NO, segments are absent from the corresponding genomic locus or their status could not be determined because of missing genomic data.
