# Supplementary material for: Genomic fossils reveal adaptation of non-autonomous pararetroviruses driven by concerted evolution of noncoding regulatory sequences
Source: PLoS Pathog. 2017 Jun 29;13(6):e1006413. doi: 10.1371/journal.ppat.1006413 (PMC5491270; doi:10.1371/journal.ppat.1006413)
Supplement: S9 Fig — The PCR strategy used to detect the presence/absence of a given ePVCVL or ePVCVL2 locus is shown at the top. Blue arrows indicate primers used for PCR detection. PCR and sequencing results are summarized in the table (+, presence; −, absence). Accessions of Oryza species used in the analysis are indicated in parentheses. Primer information is given in S6 Table. (PDF) [file ppat.1006413.s009.pdf]

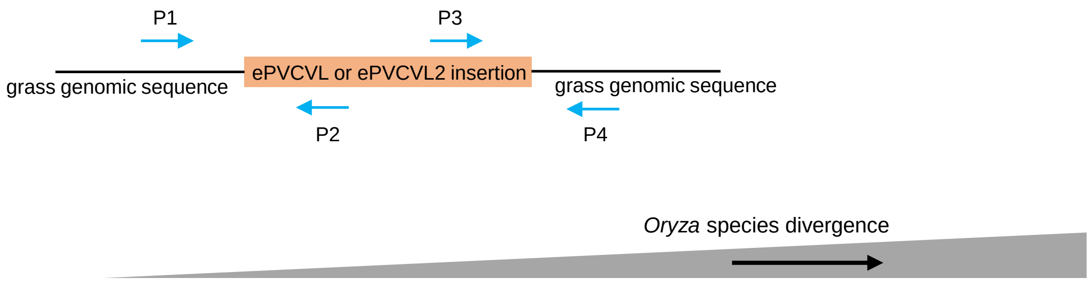

Locus	Primer pairs	FF-genome group	BB-genome group	AA-genome group				
		O. brachyantha (W1402)	*O. punctata* (W1514)	*O. meridionalis* (W2112)	*O. longistaminata* (W1413)	*O. glumaepatula* (W1169)	*O. glaberrima* (IRGC103777)	*O. sativa* (Nipponbare)
ePVCVL2-OS9	P1 + P2	-	-	-	-	+	+	+
	P3 + P4	-	-	-	-	+	+	+
ePVCVL-OS10 & ePVCVL-OS11	P1 + P2	-	-	+	+	+	+	+
	P3 + P4	-	-	+	+	+	+	+
ePVCVL2-OS5	P1 + P2	-	-	-	-	-	+	+
ePVCVL-OS3 & ePVCVL-OS4	P3 + P4	-	-	-	-	-	+	+
ePVCVL2-OS8	P1 + P4	-	-	-	-	-	-	+
ePVCVL-OS2	P1 + P4	-	-	-	-	-	-	+

S9 Fig. Genomic PCR and Sanger sequencing validation of the presence/absence of representative ePVCVL and ePVCVL2 segments (orthologous loci) in *Oryza* genomes. The PCR strategy used to detect the presence/absence of a given ePVCVL or ePVCVL2 locus is shown at the top. Blue arrows indicate primers used for PCR detection. PCR and sequencing results are summarized in the table (+, presence; −, absence). Accessions of *Oryza* species used in the analysis are indicated in parentheses. Primer information is given in S6 Table.
