# Supplementary material for: Genomic fossils reveal adaptation of non-autonomous pararetroviruses driven by concerted evolution of noncoding regulatory sequences
Source: PLoS Pathog. 2017 Jun 29;13(6):e1006413. doi: 10.1371/journal.ppat.1006413 (PMC5491270; doi:10.1371/journal.ppat.1006413)
Supplement: S10 Fig — The ML tree of IGR sequences (alignment length = 722 nt) is midpoint-rooted for display purposes. Bootstrap support values greater than 60% based on 1,000 replicates are shown as percentages above branches. Highly truncated sequences were excluded from the analysis. Scale bars represent evolutionary distances in terms of nucleotide substitutions per site. ePVCVL and ePVCVL2 sequences are indicated by green and blue, respectively (details in S3 Table). The respective orthologous sequence sets of the oldest known ePVCVL, oldest ePVCVL2, and older ePVCVL2 segments in Oryza genomes (details in S4 and S5 Tables) are indicated by purple, red, and orange backgrounds, respectively. Tanglegram of Oryza species and endogenous PRVs indicates the corresponding viral hosts. (PDF) [file ppat.1006413.s010.pdf]

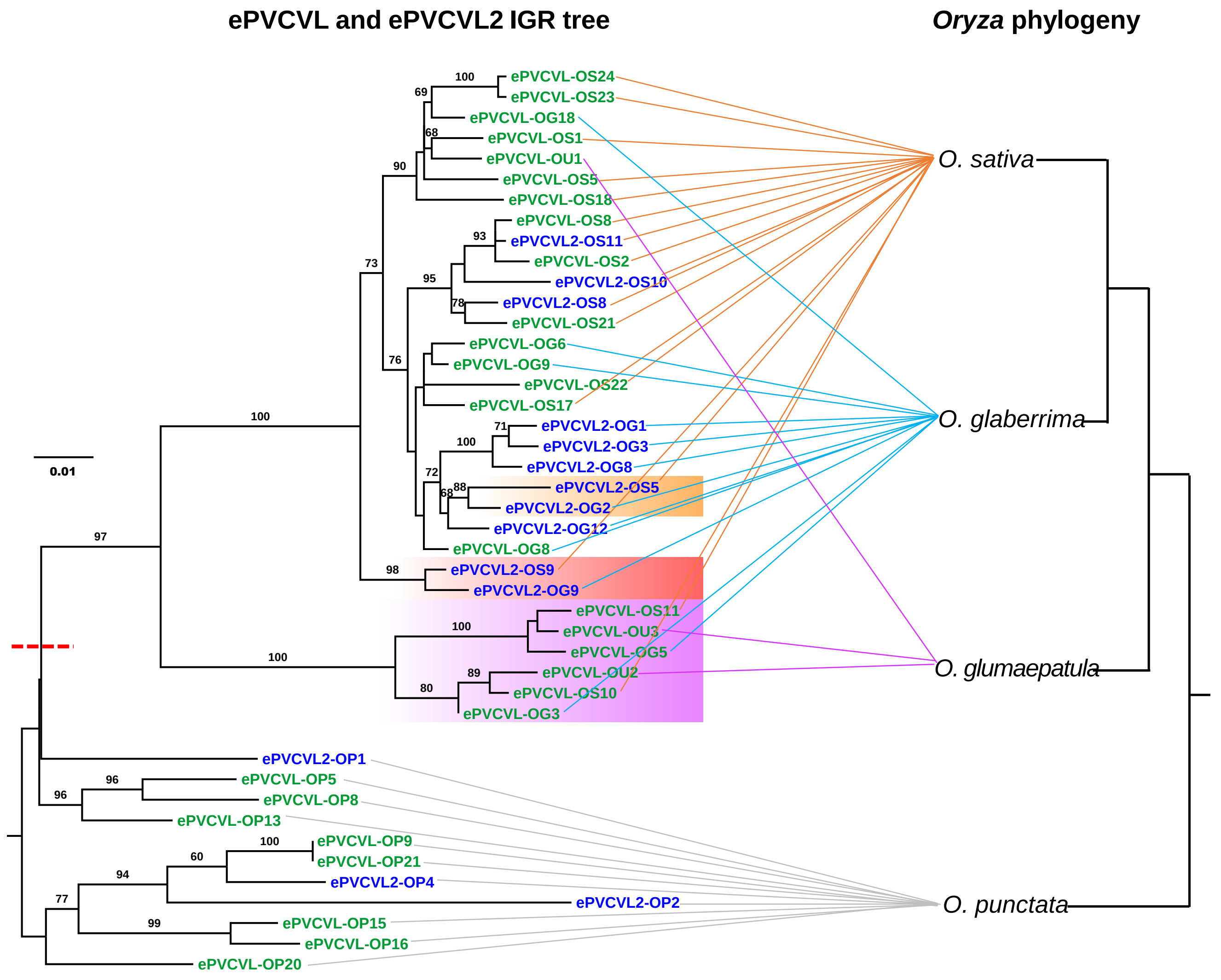

S10 Fig. Phylogenetic relationship of IGR sequences of ePVCVL and ePVCVL2 in *Oryza* genomes. The ML tree of IGR sequences (alignment length = 722 nt) is midpoint-rooted for display purposes. Bootstrap support values greater than 60% based on 1,000 replicates are shown as percentages above branches. Highly truncated sequences were excluded from the analysis. Scale bars represent evolutionary distances in terms of nucleotide substitutions per site. ePVCVL and ePVCVL2 sequences are indicated by green and blue, respectively (details in S3 Table). The respective orthologous sequence sets of the oldest known ePVCVL, oldest ePVCVL2, and older ePVCVL2 segments in *Oryza* genomes (details in S4 and S5 Tables) are indicated by purple, red, and orange backgrounds, respectively. Tanglegram of *Oryza* species and endogenous PRVs indicates the corresponding viral hosts.
