# Supplementary material for: Genomic fossils reveal adaptation of non-autonomous pararetroviruses driven by concerted evolution of noncoding regulatory sequences
Source: PLoS Pathog. 2017 Jun 29;13(6):e1006413. doi: 10.1371/journal.ppat.1006413 (PMC5491270; doi:10.1371/journal.ppat.1006413)
Supplement: S11 Fig — Pairwise alignments were generated between ePVCVL Oryza and non-Oryza groups (A) and between ePVCVL2 Oryza and non-Oryza groups (B) using consensus sequences constructed from O. sativa and S. bicolor genomes, respectively. Identical sites are indicated by black backgrounds. The relative position of the first nucleotide in each row of the alignment is given in parentheses. Red arrows, straight lines, and wavy lines indicate predicted strand discontinuity (i.e., the start of the minus-strand 5′-terminal), primer binding sites, and polypurine tracts, respectively. (PDF) [file ppat.1006413.s011.pdf]

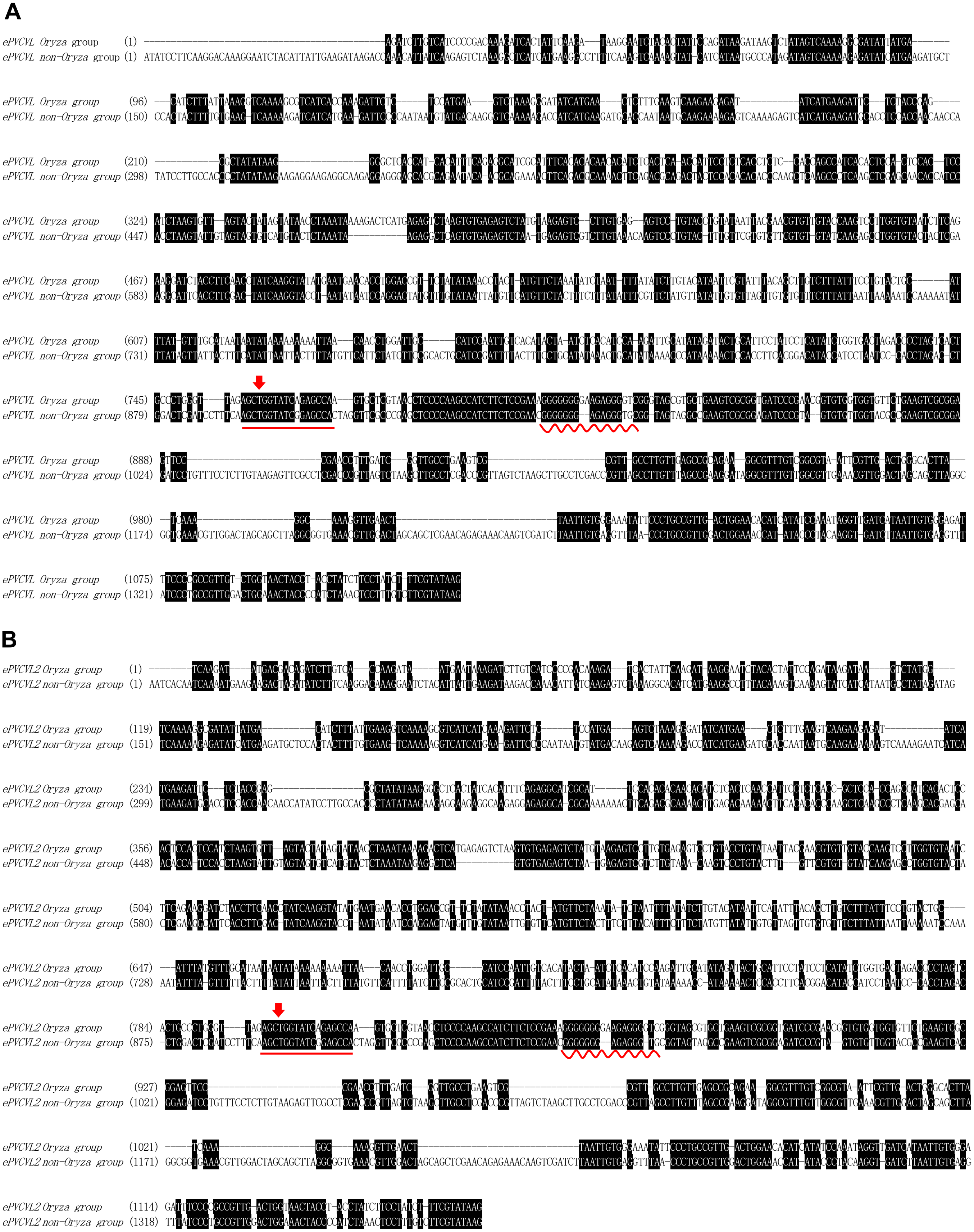

S11 Fig. Sequence comparisons of IGRs between *Oryza* and non-*Oryza* ePVCVL/ePVCVL2 groups. Pairwise alignments were generated between ePVCVL *Oryza* and non-*Oryza* groups (**A**) and between ePVCVL2 *Oryza* and non-*Oryza* groups (**B**) using consensus sequences constructed from *O. sativa* and *S. bicolor* genomes, respectively. Identical sites are indicated by black backgrounds. The relative position of the first nucleotide in each row of the alignment is given in parentheses. Red arrows, straight lines, and wavy lines indicate predicted strand discontinuity (i.e., the start of the minus-strand 5′-terminal), primer binding sites, and polypurine tracts, respectively.
