# Supplementary material for: Genomic fossils reveal adaptation of non-autonomous pararetroviruses driven by concerted evolution of noncoding regulatory sequences
Source: PLoS Pathog. 2017 Jun 29;13(6):e1006413. doi: 10.1371/journal.ppat.1006413 (PMC5491270; doi:10.1371/journal.ppat.1006413)
Supplement: S1 Table — (PDF) [file ppat.1006413.s012.pdf]

S1 Table. Information on grass genomes investigated in this study.

Genome	Common name	Data source	Data version
Oryza sativa (*japonica*)	Rice (AA-genome)	Gramene	IRGSP-1.0
Oryza glaberrima	African rice (AA-genome)	Gramene	AGI1.1
Oryza glumaepatula	Wild rice (AA-genome)	Gramene	ALNU02000000
Oryza longistaminata	Wild rice (AA-genome)	NCBI	AMDW00000000.1
Oryza meridionalis	Wild rice (AA-genome)	Gramene	ALNW00000000
Oryza punctata	Wild rice (BB-genome)	Gramene	AVCL00000000
Oryza brachyantha	Wild rice (FF-genome)	Gramene	V1.4b
Zizania latifolia	Wild rice	NCBI	ASSH00000000.1
Hordeum vulgare	Barley	Gramene	IBSC_1.0
Hordeum pubiflorum	-	NCBI	CBMN000000000.1
Triticum aestivum	Bread wheat	Gramene	IWGSP1
Triticum urartu	Wheat	Gramene	ASM34745v1
Aegilops tauschii	Goatgrass	Gramene	ASM34733v1
Brachypodium distachyon	Purple false brome	Phytozome	JGI v1.0
Zea mays	Maize	Gramene	AGPv3
Sorghum bicolor	Sorghum	Phytozome	JGI v2.1
Setaria italica	Foxtail millet	Phytozome	JGI v2.0
Setaria viridis	Green foxtail	NCGR	V02
Panicum virgatum	Switchgrass	Phytozome	V1.1
Panicum hallii	Hall's panicgrass	Phytozome	V0.5
