# Supplementary material for: Genomic fossils reveal adaptation of non-autonomous pararetroviruses driven by concerted evolution of noncoding regulatory sequences
Source: PLoS Pathog. 2017 Jun 29;13(6):e1006413. doi: 10.1371/journal.ppat.1006413 (PMC5491270; doi:10.1371/journal.ppat.1006413)
Supplement: S2 Table — (PDF) [file ppat.1006413.s013.pdf]

S2 Table. Identification of functional domains in protein sequences of ePVCVL and ePVCVL2.

Query	Domain number	Domain name/description	Note	Query residues	Subject residues	Probability (%)	*P* value	E value
ePVCVL *Oryza* group (ORF1)	PF01107	MP: Viral movement protein	MP	52-215	13-182	99.9	1.50E-30	1.80E-25
	g.40.1	Retrovirus zinc finger-like domains	CP	920-944	15-39	96.4	3.20E-08	3.80E-03
	PF02160	Peptidase_A3: Cauliflower mosaic virus peptidase	PR	1143-1277	6-144	99.7	1.40E-22	1.70E-17
ePVCVL2 *Oryza* group	PF01107	MP: Viral movement protein	MP	53-212	14-179	99.9	8.30E-31	9.80E-26
	PF02160	Cauliflower mosaic virus peptidase (A3)	PR	783-968	3-201	99.6	5.90E-20	7.00E-15
	4OL8	Reverse transcriptase/ribonuclease H	RT/RH	941-1417	13-478	100	1.40E-82	1.70E-77
ePVCVL non-*Oryza* group (ORF1)	PF01107	MP: Viral movement protein	MP	48-216	9-183	99.9	1.30E-30	1.60E-25
	g.40.1	Retrovirus zinc finger-like domains	CP	937-962	16-41	97.5	8.20E-10	9.80E-05
	PF02160	Peptidase_A3: Cauliflower mosaic virus peptidase	PR	1158-1332	6-197	99.8	1.70E-24	2.00E-19
ePVCVL2 non-*Oryza* group	PF02160	Peptidase_A3: Cauliflower mosaic virus peptidase	PR	714-896	6-201	99.4	1.40E-17	1.70E-12
	4OL8	Reverse transcriptase/ribonuclease H	RT/RH	881-1343	25-476	100	2.70E-81	3.20E-76

Domain identification was fulfilled with HHpred [38]. The hits with the best *P* value for each block of query residues are displayed. The SFKTE domain of ePVCVL and the ORF2 of ePVCVL2 are functionally unknown.
