# Supplementary material for: Genomic fossils reveal adaptation of non-autonomous pararetroviruses driven by concerted evolution of noncoding regulatory sequences
Source: PLoS Pathog. 2017 Jun 29;13(6):e1006413. doi: 10.1371/journal.ppat.1006413 (PMC5491270; doi:10.1371/journal.ppat.1006413)
Supplement: S3 Table — (PDF) [file ppat.1006413.s014.pdf]

S3 Table. Detailed information on ePVCVL and ePVCVL2 segments in grass genomes.

Grass species	Family	Segment name	Position in grass genomes				Position in virus genome		Length (bp)
			Chromosome/scaffold	Strand	Start	End	Start	End	
O. sativa	ePVCVL	ePVCVL-OS1	1	+	8840504	8847149	4	6589	6646
		ePVCVL-OS2	1	-	23908392	23902732	967	6643	5661
		ePVCVL-OS3	1	-	34125359	34121219	1488	5638	4141
		ePVCVL-OS4	1	+	34125360	34126305	459	1404	946
		ePVCVL-OS5	2	+	11304753	11311418	1	6650	6666
		ePVCVL-OS6	2	-	11312045	11311420	1	624	626
		ePVCVL-OS7	3	+	22472858	22472966	5056	5164	109
		ePVCVL-OS8	3	+	22472973	22474562	5056	6650	1590
					22474563	22474682	1	122	120
		ePVCVL-OS9	3	-	22478535	22474683	582	4442	3853
		ePVCVL-OS10	4	-	23050761	23049961	5296	6549	801
		ePVCVL-OS11	4	+	23051051	23052842	4835	6650	1792
					23052843	23052909	1	66	67
		ePVCVL-OS12	6	-	2756800	2756579	1	262	222
		ePVCVL-OS13	7	+	11608404	11609584	1821	3001	1181
		ePVCVL-OS14	8	+	25041604	25041978	6258	6650	375
		ePVCVL-OS15	8	-	25047553	25041981	271	5829	5573
		ePVCVL-OS16	9	+	20408412	20408865	62	515	454
		ePVCVL-OS17	9	+	20415024	20421219	510	6611	6196
		ePVCVL-OS18	9	-	21426023	21423510	4191	6650	2514
		ePVCVL-OS19	9	-	21431914	21430262	2545	4198	1653
		ePVCVL-OS20	9	-	21432081	21431915	5168	5350	167
		ePVCVL-OS21	10	+	8405909	8410510	2057	6650	4602
		ePVCVL-OS22	11	+	8293415	8299957	69	6646	6543
		ePVCVL-OS23	11	-	28547732	28541508	23	6650	6225
		ePVCVL-OS24	11	-	28591917	28585691	23	6650	6227
	ePVCVL2	ePVCVL2-OS1	1	-	8849149	8847147	3967	5968	2003
		ePVCVL2-OS2	2	-	11313137	11312114	3310	4333	1024
		ePVCVL2-OS3	2	-	11313900	11313138	31	800	763
		ePVCVL2-OS4	2	+	11313901	11314199	357	685	299
		ePVCVL2-OS5	5	-	20231116	20224846	64	6426	6271
		ePVCVL2-OS6	7	-	11604767	11601272	2749	6527	3496
		ePVCVL2-OS7	7	+	11604768	11605869	271	1385	1102
		ePVCVL2-OS8	7	-	27575480	27572485	3558	6558	2996
					27572484	27571939	1	561	546
		ePVCVL2-OS9	11	-	11865464	11860427	1222	6435	5038
		ePVCVL2-OS10	11	+	19845104	19848248	3385	6366	3145
		ePVCVL2-OS11	12	+	19505111	19511369	42	6356	6259
O. glaberrima	ePVCVL	ePVCVL-OG1	1	-	24848084	24847398	4731	5417	687
		ePVCVL-OG2	1	-	24850826	24849905	2675	3596	922
		ePVCVL-OG3	4	-	15508021	15507736	6265	6549	286
		ePVCVL-OG4	4	-	15508460	15508338	5314	5437	123
		ePVCVL-OG5	4	+	15508778	15510896	4835	6650	2119
					15510897	15510963	1	66	67
		ePVCVL-OG6	4	-	26265352	26260099	1663	6650	5254
		ePVCVL-OG7	4	+	26265353	26265878	1048	1592	526
		ePVCVL-OG8	6	+	16933152	16940095	54	6650	6944
					16940096	16940152	1	57	57
		ePVCVL-OG9	10	+	6511230	6516580	1434	6593	5351
		ePVCVL-OG10	10	+	8397190	8401147	734	4471	3958

		ePVCVL-OG11	10	+	8403814	8405594	4466	6246	1781
		ePVCVL-OG12	11	+	6585334	6587452	2334	4453	2119
		ePVCVL-OG13	11	-	15893636	15893277	4663	5023	360
		ePVCVL-OG14	11	+	15893637	15894763	1170	2312	1127
		ePVCVL-OG15	11	+	15895817	15897227	2708	4118	1411
		ePVCVL-OG16	12	-	13437088	13433323	1488	4597	3766
		ePVCVL-OG17	12	+	13437089	13437510	459	891	422
		ePVCVL-OG18	12	+	13437516	13438725	5418	6611	1210
		ePVCVL-OG19	Oglab12_unplaced047	+	58	4574	357	5062	4517
	ePVCVL2	ePVCVL2-OG1	4	-	26260103	26253603	15	6558	6501
		ePVCVL2-OG2	5	-	15500612	15494086	64	6420	6527
		ePVCVL2-OG3	7	+	7758507	7764924	1	6524	6418
		ePVCVL2-OG4	9	+	16969612	16970506	279	1173	895
		ePVCVL2-OG5	9	+	16970503	16971982	4749	6229	1480
		ePVCVL2-OG6	10	-	8397098	8392335	1568	6154	4764
		ePVCVL2-OG7	11	-	6045997	6045480	6046	6558	518
					6045479	6044654	1	1285	826
		ePVCVL2-OG8	11	-	6592706	6587472	1286	6558	5235
		ePVCVL2-OG9	11	-	9224687	9219391	1222	6435	5297
		ePVCVL2-OG10	11	+	15896886	15897190	2706	3010	305
		ePVCVL2-OG11	11	-	15899867	15899177	1518	2241	691
		ePVCVL2-OG12	Oglab02_unplaced013	-	19352	17967	5146	6521	1386
		ePVCVL2-OG13	Oglab02_unplaced013	-	22131	20365	1570	3370	1767
O. glumaepatula	ePVCVL	ePVCVL-OU1	3	+	4729510	4730670	5485	6650	1161
		ePVCVL-OU2	4	-	20425951	20425145	5296	6549	807
		ePVCVL-OU3	4	+	20426253	20428023	4843	6650	1771
					20428024	20428093	1	66	70
		ePVCVL-OU4	6	-	25698260	25697867	250	643	394
		ePVCVL-OU5	12	+	21618343	21619520	3736	4914	1178
		ePVCVL-OU6	12	+	21626409	21626529	6530	6650	121
	ePVCVL2	ePVCVL2-OU1	1	-	25261149	25260838	6238	6543	312
		ePVCVL2-OU2	10	-	15996112	15995870	5351	5593	243
		ePVCVL2-OU3	11	-	11666533	11665031	1222	2726	1503
O. longistaminata	ePVCVL	ePVCVL-OL1	Scaffold122663	-	42442	42094	1164	1512	349
		ePVCVL-OL2	Scaffold136348	-	10166	8367	4140	6549	1800
		ePVCVL-OL3	Scaffold136348	+	10226	11234	5763	6650	1009
					11235	11298	1	75	64
	ePVCVL2	ePVCVL2-OL1	Scaffold126431	+	170647	170724	6452	6529	78
		ePVCVL2-OL2	Scaffold136293	-	6470	6407	6095	6162	64
O. meridionalis	ePVCVL	ePVCVL-OM1	1	+	843164	844272	5006	5785	1109
		ePVCVL-OM2	1	-	19176727	19176596	3867	3998	132
		ePVCVL-OM3	2	+	19424534	19424933	5150	5410	400
		ePVCVL-OM4	3	+	2366827	2368542	2080	3410	1716
		ePVCVL-OM5	3	+	2369641	2370890	3258	4414	1250
		ePVCVL-OM6	8	+	9592885	9593588	4729	5204	704
		ePVCVL-OM7	8	-	10030062	10029035	4510	5344	1028
		ePVCVL-OM8	9	+	3194279	3194422	6136	6279	144
	ePVCVL2	ePVCVL2-OM1	4	-	23170204	23169068	4527	5663	1137
		ePVCVL2-OM2	6	+	11359819	11361254	2628	3552	1436
		ePVCVL2-OM3	7	-	18949381	18949008	4536	4909	374
		ePVCVL2-OM4	8	-	5997777	5996654	617	1740	1124
		ePVCVL2-OM5	8	-	5999649	5999224	5834	6138	426
		ePVCVL2-OM6	8	+	9583173	9583690	4196	4579	518
		ePVCVL2-OM7	8	-	10030167	10030053	4129	4243	115
		ePVCVL2-OM8	9	-	11799894	11798813	3530	4249	1082

		ePVCVL2-OM9	11	-	7758285	7758179	1	108	107
		ePVCVL2-OM10	11	+	7758637	7759163	2056	2447	527
O. punctata	ePVCVL	ePVCVL-OP1	2	-	18174237	18173741	1002	1500	497
		ePVCVL-OP2	2	+	18174238	18174792	1520	2085	555
		ePVCVL-OP3	2	+	18174893	18175461	3969	4537	569
		ePVCVL-OP4	2	+	18175564	18175698	1551	1685	135
		ePVCVL-OP5	2	+	18175703	18178269	4053	6628	2567
		ePVCVL-OP6	2	-	18178605	18178270	31	369	336
		ePVCVL-OP7	2	+	18178606	18180215	357	1988	1610
		ePVCVL-OP8	2	+	18180216	18182535	4359	6650	2320
		ePVCVL-OP9	3	-	1877451	1870791	1	6629	6661
		ePVCVL-OP10	3	-	1877899	1877452	6228	6650	448
		ePVCVL-OP11	4	-	11094038	11089082	44	5005	4957
		ePVCVL-OP12	7	-	14019246	14018987	3763	4022	260
		ePVCVL-OP13	7	+	14019291	14021601	4347	6650	2311
					14021602	14021994	1	408	393
		ePVCVL-OP14	7	-	14022570	14022140	3935	4365	431
		ePVCVL-OP15	9	+	2488988	2494894	476	6650	5907
		ePVCVL-OP16	9	+	2508244	2509198	5707	6650	955
					2509199	2509685	1	490	487
		ePVCVL-OP17	9	-	2510214	2509778	6191	6646	437
		ePVCVL-OP18	9	-	2511022	2510216	5703	6471	807
		ePVCVL-OP19	9	+	2511023	2513219	1330	3521	2197
		ePVCVL-OP20	9	+	2513220	2515543	4196	6514	2324
		ePVCVL-OP21	12	-	17241666	17239641	4690	6629	2026
		ePVCVL-OP22	12	-	17241795	17241667	1758	1891	129
		ePVCVL-OP23	12	-	17242162	17241796	2142	2513	367
	ePVCVL2	ePVCVL2-OP1	1	+	36462406	36468381	497	6529	5976
		ePVCVL2-OP2	7	-	14512232	14518685	56	6548	6454
		ePVCVL2-OP3	12	+	17242165	17243967	1	1803	1803
		ePVCVL2-OP4	12	+	17245498	17250240	1798	6539	4743
S. bicolor	ePVCVL	ePVCVL-SB1	3	-	293657	286801	1	6879	6857
		ePVCVL-SB2	3	-	18263770	18263397	4846	5222	374
		ePVCVL-SB3	3	-	18270253	18267703	4321	6880	2551
		ePVCVL-SB4	4	+	46230217	46237235	13	6890	7019
		ePVCVL-SB5	5	-	12982623	12982416	6667	6890	208
		ePVCVL-SB6	5	+	13007912	13013637	1152	6890	5726
		ePVCVL-SB7	5	+	13013665	13020548	1	6872	6884
		ePVCVL-SB8	5	-	13715088	13710895	1955	6179	4194
		ePVCVL-SB9	5	+	33532220	33538691	196	6759	6472
		ePVCVL-SB10	5	+	47930070	47936859	10	6796	6790
		ePVCVL-SB11	5	-	58530701	58530474	5519	5751	228
		ePVCVL-SB12	5	+	58530702	58531602	1	926	901
		ePVCVL-SB13	5	+	58531603	58536533	1960	6890	4931
		ePVCVL-SB14	5	+	58536624	58537273	1	674	650
		ePVCVL-SB15	5	-	58541750	58537274	569	5057	4477
		ePVCVL-SB16	6	+	31383876	31384127	6628	6890	252
		ePVCVL-SB17	6	+	31384129	31388146	2887	6890	4018
					31388147	31391037	1	2965	2891
		ePVCVL-SB18	6	+	37580566	37581109	6352	6890	544
		ePVCVL-SB19	6	+	37581137	37587995	1	6890	6859
					37587996	37588062	1	68	67
		ePVCVL-SB20	6	-	44430825	44423973	4	6890	6853
		ePVCVL-SB21	7	+	36892423	36897609	1	5688	5187
		ePVCVL-SB22	7	-	36898552	36897755	5324	6123	798

		ePVCVL-SB23	7	-	36901720	36898553	2513	6140	3168
		ePVCVL-SB24	Super_19	+	790000	792947	677	3641	2948
		ePVCVL-SB25	Super_19	+	793795	795128	5082	6429	1334
		ePVCVL-SB26	Super_19	+	795337	796253	4127	5041	917
		ePVCVL-SB27	Super_19	+	796354	796769	6461	6890	416
		ePVCVL-SB28	Super_19	+	796771	800729	2887	6867	3959
	ePVCVL2	ePVCVL2-SB1	1	+	54922322	54928778	1	6544	6457
					54928779	54928805	1	27	27
		ePVCVL2-SB2	4	+	16773368	16779949	1	6499	6582
		ePVCVL2-SB3	6	+	37559217	37562676	123	3583	3460
		ePVCVL2-SB4	6	-	37563756	37562692	5027	6029	1065
		ePVCVL2-SB5	6	-	37571348	37566378	22	5032	4971
		ePVCVL2-SB6	10	-	16463710	16457225	3	6544	6486
					16457224	16457110	1	115	115
		ePVCVL2-SB7	10	+	16463785	16463938	6198	6351	154
P. virgatum	ePVCVL	ePVCVL-PV1	3b	-	5042450	5042142	1991	2322	309
		ePVCVL-PV2	7a	+	35051553	35051689	6487	6635	137
					35052031	35054066	1	2699	2036
		ePVCVL-PV3	7a	+	39593523	39594633	2684	3795	1111
		ePVCVL-PV4	7a	-	39595884	39595051	1146	1969	834
		ePVCVL-PV5	7a	-	39602918	39601951	1	1079	968
		ePVCVL-PV6	Contig04516	-	10517	6085	2054	6651	4433
		ePVCVL-PV7	Contig171646	-	646	389	1414	1675	258
		ePVCVL-PV8	Contig171646	-	953	716	404	639	238
		ePVCVL-PV9	Contig171646	-	1469	1173	333	618	297
		ePVCVL-PV10	Contig171646	-	2090	1972	31	151	119
	ePVCVL2	ePVCVL2-PV1	7a	-	40139014	40138744	3240	3510	271
		ePVCVL2-PV2	7a	+	40139022	40141657	3273	5903	2636
		ePVCVL2-PV3	8b	+	47417775	47417980	336	541	206

Each of the ePVCVL and ePVCVL2 segments was mapped to the respective virus genomes of ePVCVL and ePVCVL2 reconstructed from the *O. sativa* (*japonica*) genome, or to those constructed from the *S. bicolor* genome. Considering the assemble level of *O. longistaminata* genomic data we used, the segments shorter than 100 bp were also listed.
