# Supplementary material for: Genomic fossils reveal adaptation of non-autonomous pararetroviruses driven by concerted evolution of noncoding regulatory sequences
Source: PLoS Pathog. 2017 Jun 29;13(6):e1006413. doi: 10.1371/journal.ppat.1006413 (PMC5491270; doi:10.1371/journal.ppat.1006413)
Supplement: S4 Table — (PDF) [file ppat.1006413.s015.pdf]

S4 Table. Mapping of ePVCVL and ePVCVL2 loci in the *O. sativa* genome to other *Oryza* genomes.

Information of ePVCVL and ePVCVL2 loci in the *O. sativa* genome		Mapping into the other *Oryza* genomes[c]					Remark
**Locus number (position)[a]**	**Segments[b]**	***O. glaberrima***	***O. glumaepatula***	***O. longistaminata***	***O. meridionalis***	***O. punctata***	
Locus1 (1:8840504-8849149)	ePVCVL-OS1, ePVCVL2-OS1	NO (1:6923482-6923778)	NO (1:10192607-10194752)	NO (Scaffold140566:≈267573)	NO (1:≈8993855)	NO (1:≈8415586)	specific
Locus2 (1:23902732-23908392)	ePVCVL-OS2	NO (1:16686414-16686464)	NO (1:26043345-26062879)	NO (Scaffold133964:≈230019)	NO (1:21706486-21706494)	NO (1:≈25718279)	specific
Locus3 (1:34121219-34126305)	ePVCVL-OS3, ePVCVL-OS4	YES (1:24847398-24850826)	NO (1:37035535-37039966)	NO (Scaffold127363:20467-20936)	NO (1:≈32368856)	NO (1:≈36538050)	shared
Locus4 (2:11304685-11314199)	ePVCVL-OS5, ePVCVL-OS6, ePVCVL2-OS2, ePVCVL2-OS3, ePVCVL2-OS4	NO (2:≈9990278)	NO (2:≈10786295)	NA	NO (2:≈12965281)	NO (2:≈11085428)	specific
Locus5 (3:22472858-22478535)	ePVCVL-OS7, ePVCVL-OS8, ePVCVL-OS9	NO (3:21127466-21144299)	NO (3:24188491-24207759)	NO (Scaffold133936:≈11952)	NO (3:23820900-23831718)	NO (3:≈24683602)	specific
Locus6 (4:23049961-23052909)	ePVCVL-OS10, ePVCVL-OS11	YES (4:15507736-15510963)	YES (4:20425145-20428093)	YES(Scaffold136348:8367-11276)	YES (by PCR)	NO (4:20701665-20702064)	shared
Locus7 (5:20224846-20231116)	ePVCVL2-OS5	YES (5:15494086-15500612)	NO (5:20226666-20226726)	NA	NO (5:≈17927496)	NA	shared
Locus8 (6:2756579-2756800)	ePVCVL-OS12	NA	NA	NA	NA	NA	undetermined
Locus9 (7:11601272-11609584)	ePVCVL-OS13, ePVCVL2-OS6, ePVCVL2-OS7	NO (7:≈9685762)	NO (7:≈10281454)	NO (Scaffold135424:≈13135)	NA	NO (7:≈12715201)	specific
Locus10 (7:27571939-27575480)	ePVCVL2-OS8	NO (7:19735543-19735565)	NO (7:26263270-26263297)	NO (Scaffold136766:46190-52571)	NO (7:24129203-24129252)	NO (7:29102683-29102710)	specific
Locus11 (8:25041604-25047553)	ePVCVL-OS14, ePVCVL-OS15	NO (8:18879456-18879465)	NO (8:23608448-23608461)	NO (Scaffold133581:28131-28144)	NO (8:19905457-19905466)	NA	specific
Locus12 (9:20408412-20408865)	ePVCVL-OS16	NO (9:≈15164188)	NO (9:≈20532533)	NO (Scaffold127286:≈53721)	NO (9:≈16147417)	NO (9:≈23782384)	specific
Locus13 (9:20415024-20421219)	ePVCVL-OS17	NO (9:≈15166306)	NO (9:≈20549604)	NO (Scaffold134818:≈47966)	NO (9:≈16163653)	NO (9:≈23789563)	specific
Locus14 (9:21423506-21432081)	ePVCVL-OS18, ePVCVL-OS19, ePVCVL-OS20	NA	NO (9:≈21601883)	NO (Scaffold136665:≈296524)	NO (9:≈17419017)	NA	undetermined
Locus15 (10:8405909-8410513)	ePVCVL-OS21	NO (10:≈12586160)	NO (10:7693053-7693094)	NO (Scaffold134487:≈52955)	NA	NA	specific
Locus16 (11:8293415-8299957)	ePVCVL-OS22	NO (11:≈6580050)	NO (11:≈8208302)	NA	NA	NA	specific
Locus17 (11:11860427-11865464)	ePVCVL2-OS9	YES (11:9219391-9224687)	YES (11:11665031-11666533)	NO (Scaffold135116:≈8865)	NO (11:≈12525767)	NO (by PCR)	shared
Locus18 (11:19845104-19848248)	ePVCVL2-OS10	NO (11:≈15223836)	NO (11:≈19127289)	NO (Scaffold138725:≈12970)	NO (11:16885801-16885858)	NA	specific
Locus19 (11:28541504-28591917)	ePVCVL-OS23, ePVCVL-OS24	NO (6:≈11963074)	NA	NO (Scaffold127573:63004-63012)	NA	NO (7:≈12374530)	specific
Locus20 (12:19505111-19511369)	ePVCVL2-OS11	NO (Oglab12_unplaced156:40610-41300)	NO (12:19470470-19470551)	NO (Scaffold124318:≈104739)	NO (12:≈13183476)	NO (12:≈20578764)	specific

[a]The segments close to each other (interval < 5kb) in the chromosomes are taken as a locus in the mapping analysis (locus positions are provided in the brackets).

[b]Names of the segments within a locus.

[c]Mapping results are of three kinds: YES, presence of a orthologous insertion at the corresponding locus (positions of orthologous insertions are shown in the brackets); NO, absence of a orthologous insertion at the corresponding locus (predicted position or range of empty sites are shown in the brackets); NA, not available due to a mass of uncharacterized bases (missing data), or a large deletion or rearrangement at the corresponding locus.
