# Supplementary material for: Genomic fossils reveal adaptation of non-autonomous pararetroviruses driven by concerted evolution of noncoding regulatory sequences
Source: PLoS Pathog. 2017 Jun 29;13(6):e1006413. doi: 10.1371/journal.ppat.1006413 (PMC5491270; doi:10.1371/journal.ppat.1006413)
Supplement: S5 Table — (PDF) [file ppat.1006413.s016.pdf]

S5 Table. Mapping of ePVCVL and ePVCVL2 loci in the genomes of *O. glaberrima*, *O. glumaepatula*, *O. longistaminata*, *O. meridionalis* and *O. punctata* to the genome of *O. sativa*.

Genome	Information of ePVCVL and ePVCVL2 loci in the genomes		Mapping into the *O. sativa* genome[c]	Remark
	Locus number (position)[a]	Segments[b]		
O. glaberrima	Locus1 (1:24847398-24850826)	ePVCVL-OG1, ePVCVL-OG2	YES (1:34121219-34126305)	shared
	Locus2 (4:15507736-15510963)	ePVCVL-OG3, ePVCVL-OG4, ePVCVL-OG5	YES (4:23049961-23052909)	shared
	Locus3 (4:26253608-26265878)	ePVCVL-OG6, ePVCVL-OG7, ePVCVL2-OG1	NO (4:35405701-35405717)	specific
	Locus4 (5:15494086-15500612)	ePVCVL2-OG2	YES (5:20224846-20231116)	shared
	Locus5 (6:16933152-16940152)	ePVCVL-OG8	NO (6:22693382-22693889)	specific
	Locus6 (7:7758503-7764924)	ePVCVL2-OG3	NO (7:9011784-9011913)	specific
	Locus7 (9:16969612-16971982)	ePVCVL2-OG4, ePVCVL2-OG5	NA	undetermined
	Locus8 (10:6511230-6516569)	ePVCVL-OG9	A(10:≈8419468)	specific
	Locus9 (10:8392335-8405594)	ePVCVL-OG10, ePVCVL-OG11, ePVCVL2-OG6	NO (10:10945698-10945702)	specific
	Locus10 (11:6044654-6045997)	ePVCVL2-OG7	NO (11:7617002-7617023)	specific
	Locus11 (11:6585334-6592706)	ePVCVL-OG12, ePVCVL2-OG8	NO (11:≈8377686)	specific
	Locus12 (11:9219391-9224687)	ePVCVL2-OG9	YES (11:11860427-11865464)	shared
	Locus13 (11:15893277-15899867)	ePVCVL-OG13, ePVCVL-OG14, ePVCVL-OG15, ePVCVL2-OG10, ePVCVL2-OG11	NO (11:20971323-20971750)	specific
	Locus14 (12:13433323-13438696)	ePVCVL-OG16, ePVCVL-OG17, ePVCVL-OG18	NA	undetermined
	Locus15 (Oglab02_unplaced013:17967-22131)	ePVCVL2-OG12, ePVCVL2-OG13	NA	undetermined
	Locus16 (Oglab12_unplaced047:1-4574)	ePVCVL-OG19	NA	undetermined
O. glumaepatula	Locus1 (1:25260891-25261149)	ePVCVL2-OU1	NO (1:23156362-23156379)	specific
	Locus2 (3:4729510-4730602)	ePVCVL-OU1	NO (3:4680875-4688150)	specific
	Locus3 (4:20425145-20428093)	ePVCVL-OU2, ePVCVL-OU3	YES (4:23049961-23052909)	shared
	Locus4 (6:25697867-25698260)	ePVCVL-OU4	NO (6:25598182-23052912)	specific
	Locus5 (10:15995870-15996112)	ePVCVL2-OU2	NO (10:16866946-16867588)	specific
	Locus6 (11:11665031-11666533)	ePVCVL2-OU3	YES (11:11860427-11865464)	shared
	Locus7 (12:21618343-21619520)	ePVCVL-OU5	NA	undetermined
	Locus8 (12:21626409-21626532)	ePVCVL-OU6	NO (12:≈22229076)	specific
O. longistaminata	Locus1 (Scaffold122663:42094-42442)	ePVCVL-OL1	NO (11:≈18951159)	specific
	Locus2 (Scaffold136348:8367-11298)	ePVCVL-OL2, ePVCVL-OL3	YES (4:23049961-23052931)	shared
	Locus3 (Scaffold126431:170647-170724)	ePVCVL2-OL1	NO (3:≈26353077)	specific
	Locus4 (Scaffold136293:6407-6470)	ePVCVL2-OL2	NO (7:4570128-4570212)	specific
O. meridionalis	Locus1 (1:843164-844272)	ePVCVL-OM1	NO (1:≈1361354)	specific
	Locus2 (1:19176596-19176727)	ePVCVL-OM2	NO (1:20650545-20652372)	specific
	Locus3 (2:19424534-19424933)	ePVCVL-OM3	NO (2:≈18184236)	specific
	Locus4 (3:2366827-2370890)	ePVCVL-OM4, ePVCVL-OM5	NO (3:≈1981605)	specific
	Locus5 (4:23169068-23170204)	ePVCVL2-OM1	NO (4:≈28177610)	specific
	Locus6 (6:11359819-11361254)	ePVCVL2-OM2	NO (6:9374355-9374493)	specific
	Locus7 (7:18949008-18949381)	ePVCVL2-OM3	NO (7:≈22528997)	specific
	Locus8 (8:5996654-5999649)	ePVCVL2-OM4, ePVCVL2-OM5	NO (8:6195297-6195410)	specific
	Locus9 (8:9583173-9583690)	ePVCVL2-OM6	NA	undetermined
	Locus10 (8:9592885-9593588)	ePVCVL-OM6	NA	undetermined
	Locus11 (8:10029035-10030167)	ePVCVL-OM7, ePVCVL2-OM7	NO (8:≈13106697)	specific
	Locus12 (9:3194279-3194422)	ePVCVL-OM8	NO (9:≈4554048)	specific
	Locus13 (9:11798813-11799894)	ePVCVL2-OM8	NO (9:14820313-14820314)	specific
	Locus14 (11:7758179-7759163)	ePVCVL2-OM9, ePVCVL2-OM10	NO (11:≈13741299)	specific
O. punctata	Locus1 (1:36462406-36468381)	ePVCVL2-OP1	NO (1:≈34032877)	specific
	Locus2 (2:18173741-18182535)	ePVCVL-OP1, ePVCVL-OP2, ePVCVL-OP3, ePVCVL-OP4, ePVCVL-OP5, ePVCVL-OP6, ePVCVL-OP7, ePVCVL-OP8	NO (2:≈16229047)	specific
	Locus3 (3:1870791-1877899)	ePVCVL-OP9, ePVCVL-OP10	NO (3:1950314-1952016)	specific
	Locus4 (4:11089082-11094038)	ePVCVL-OP11	NA	undetermined
	Locus5 (7:14018987-14022570)	ePVCVL-OP12, ePVCVL-OP13, ePVCVL-OP14	NO (7:≈13073519)	specific
	Locus6 (7:14512232-14518685)	ePVCVL2-OP2	NO (7:≈13488047)	specific
	Locus7 (9:2488988-2494897)	ePVCVL-OP15	NA	undetermined
	Locus8 (9:2508244-2515543)	ePVCVL-OP16, ePVCVL-OP17, ePVCVL-OP18, ePVCVL-OP19, ePVCVL-OP20	NA	undetermined
	Locus9 (12:17239641-17250240)	ePVCVL-OP21, ePVCVL-OP22, ePVCVL-OP23, ePVCVL2-OP3, ePVCVL2-OP4	NO (12:≈19505111)	specific

[a]The segments close to each other (interval < 5kb) in the chromosomes are taken as a locus in the mapping analysis (locus positions are provided in the brackets).

[b]Names of the segments within a locus.

[c]Mapping results are of three kinds: YES, presence of a orthologous insertion at the corresponding locus (positions of orthologous insertions are shown in the brackets); NO, absence of a orthologous insertion at the corresponding locus (predicted position or range of empty sites are shown in the brackets); NA, not available due to a mass of uncharacterized bases (missing data), or a large deletion or rearrangement at the corresponding locus.
