# Supplementary material for: Genomic fossils reveal adaptation of non-autonomous pararetroviruses driven by concerted evolution of noncoding regulatory sequences
Source: PLoS Pathog. 2017 Jun 29;13(6):e1006413. doi: 10.1371/journal.ppat.1006413 (PMC5491270; doi:10.1371/journal.ppat.1006413)
Supplement: S6 Table — (PDF) [file ppat.1006413.s017.pdf]

S6 Table. Information on the primers used in this study.

Target	Primer name	Primer sequence (from 5' to 3')	Remark
Orthologous ePVCVL2-OS9	eRPV1-st6-1b	TGGTATGTGGCAATTGGATGG	forward
	eRPV1-st6-2	TAAAGATCCTGTCATCCCCGA	reverse
	eRPV1-st6-2b	ATTGCAAGGCAGCAAAAGAT	reverse
	eRPV1-st6-3	CCCTTTTGCTGTTCGTTGTTGGC	forward
	eRPV1-st6-4	TGCTCACCTCCCCACCTCGC	reverse
Orthologous ePVCVL-OS10 & ePVCVL-OS11	eRPV2-st6-1a	CCACTGGGTATCAGCGTTCAATC	forward
	eRPV2-st6-2	CCTATATAAGGGGCTCAGGAACAC	reverse
	eRPV2-st6-M1	TAGACTCTCACACTTAGACTTTC	forward
	eRPV2-st6-M2a	CGGTCTAGGTGTTCATTCATATAC	reverse
	eRPV2-st6-M2b	CTTGATAGGTTGAAGGTAGATCC	reverse
	eRPV2-st6-3	GAAGACTTATGAAAGTCAAGTGTGAG	forward
	eRPV2-st6-4	GCATATCTTGCAAGAAAATCGTAAGTC	reverse
Orthologous ePVCVL2-OS5	eRPV1-ga2-1	TTGAGGGGCGATTCAATTAG	forward
	eRPV1-ga2-2	GGCTCACCATCACACTTCGG	reverse
Orthologous ePVCVL-OS3 & ePVCVL-OS4	eRPV2-ga1-3	CACTGGTTGTCTCCCATTCTATAG	forward
	eRPV2-ga1-4	CGTTCGTTCGCTGTAAATCAG	reverse
Orthologous ePVCVL2-OS8	eRPV1-st5-1	GCAATTAGGCTCCACGAAGGATTTA	forward
	eRPV1-st5-4	GTTGGCTCCTGGCCCTTCACAC	reverse
Orthologous ePVCVL-OS2	eRPV2-st2-1	GAGAAATCGGAGTTCATCGTCT	forward
	eRPV2-st2-4	CGTTTCCGTGTAAGGGCTAC	reverse
